# Post-translational modification of CDK1–STAT3 signaling by fisetin suppresses pancreatic cancer stem cell properties

**DOI:** 10.1186/s13578-023-01118-z

**Published:** 2023-09-24

**Authors:** Xiaodong Xu, Yimin Ding, Junbin Jin, Chengjie Xu, Wenyi Hu, Songtao Wu, Guoping Ding, Rui Cheng, Liping Cao, Shengnan Jia

**Affiliations:** 1 Department of General Surgery, Sir Run Run Shaw Hospital, School of Medicine, Zhejiang University, Hangzhou, 310000 China; 2 Innovation Center for Minimally Invasive Technique and Device, Zhejiang University, Hangzhou, 310000 Zhejiang China; 3 State Key Laboratory of Food Science and Technology, Jiangnan University, 1800 Lihu Avenue, Wuxi, 214122 China; 4 General Surgery, Cancer Center, Department of Colorectal Surgery, Zhejiang Provincial People’s Hospital (Affiliated People’s Hospital, Hangzhou Medical College), Hangzhou, 310014 Zhejiang China

**Keywords:** CDK1, Fisetin, Cancer stem cells, STAT3, Pancreatic ductal adenocarcinoma

## Abstract

**Background:**

Pancreatic cancer stem cells (CSCs) promote pancreatic ductal adenocarcinoma (PDAC) tumorigenesis and chemoresistance. Cyclin-dependent kinase 1 (CDK1) plays an important role in tumor initiation in other tumors, but the function of CDK1 in PDAC remains unclear. Fisetin is a bioactive flavonoid with anti-tumor properties in multiple tumors, while its function in CSCs remains elusive.

**Results:**

In this study, we demonstrated that CDK1 was correlated with prognosis and was highly expressed in pancreatic cancer tissue and gemcitabine-resistant cells. Silencing *CDK1* impaired tumor stemness and reduced a subset of CSCs. We found that fisetin blocked the kinase pocket domain of CDK1 and inhibited pancreatic CSC characteristics. Using acetylation proteomics analysis and phosphorylation array assay, we confirmed that fisetin reduced CDK1 expression and increased CDK1 acetylation at lysine 33 (K33), which resulted in the suppression of CDK1 phosphorylation. Silencing *CDK1* or *STAT3* suppressed tumor stemness properties, while overexpressing CDK1 or STAT3 showed the opposite effect. Mutation or acetylation of CDK1 at K33 weakened STAT3 phosphorylation at Y705, impairing the expression of stem-related genes and pancreatic cancer stemness. In addition, lack of histone deacetylase 3 (HDAC3), which deacetylates CDK1, contributed to weakening STAT3 phosphorylation by regulating the post-translational modification of CDK1, thereby decreasing the stemness of PDAC. Moreover, our results revealed that fisetin enhanced the effect of gemcitabine through eliminating a subpopulation of pancreatic CSCs by inhibiting the CDK1–STAT3 axis in vitro and in vivo.

**Conclusion:**

Our findings highlight the role of post-translational modifications of CDK1–STAT3 signaling in maintaining cancer stemness of PDAC, and indicated that targeting the CDK1–STAT3 axis with inhibitors such as fisetin is a potential therapeutic strategy to diminish drug resistance and eliminate PDAC.

**Supplementary Information:**

The online version contains supplementary material available at 10.1186/s13578-023-01118-z.

## Background

Pancreatic adenocarcinoma (PDAC) is an extremely malignant tumor characterized by its high lethality and low rates of early diagnosis and radical resection [1]. It is the fourth leading cause of cancer-related death, with an upward trend worldwide [1, 2]. PDAC patients are frequently resistant to chemotherapy and radiotherapy, with 5-year survival rates of 9% [1, 3].

Cancer stem cells (CSCs) are a subpopulation of highly plastic stem-like cells in tumors that have tumor-initiating ability as well as powerful self-renewal capacity. Increasing evidence suggests that CSCs promote tumorigenesis, metastasis, recurrence, and drug resistance [4–7]. Therefore, targeting pancreatic CSCs with inhibitors might have therapeutic potential in restoring drug sensitivity and eradicating pancreatic cancer.

Cyclin-dependent kinases (CDKs) are a family of protein kinases that govern the mammalian cell cycle [8]. Dysregulation of CDKs not only accelerates tumor growth but also induces sustained or spontaneous proliferation of cancer cells. Downregulation of CDKs may lead to defects in specific tissue homeostasis, while hyperactivation of CDKs may promote tumor development by inducing unplanned cell division in stem or progenitor cells [9]. Recent studies have revealed that CDK1 plays an important role in tumor initiation and stem cell pluripotency [10–12]. Menon et al. [10] found that CDK1 binds to Sox2 and regulates its transcriptional activity, thereby promoting its tumor initiating potential. Heo et al. [11] demonstrated that CDK1‐mediated TFCP2L1 phosphorylation is associated with tumorigenic stemness features and pluripotency in bladder cancer, while Michowski et al. [12] revealed that CDK1 functions to phosphorylate various epigenetic regulators and maintain the epigenetic identity of embryonic stem cells.

Fisetin is a naturally derived bioactive flavonoid that is found in a variety of vegetables and fruits. Fisetin has anti-tumor properties in multiple cancer types such as prostate cancer, lung cancer, melanoma, breast cancer, and colon cancer [13]. We previously found that fisetin improves the effect of the first-line chemotherapy drug gemcitabine and inhibits the proliferation of PDAC cells [14, 15]. An increasing body of evidence has revealed that pancreatic CSCs (PCSCs) are responsible for driving tumor growth and relapse after chemotherapy, and PDAC cells accumulate resistance to gemcitabine due to their enhanced stemness resulting from the treatment with gemcitabine [7, 16, 17].

In this study, we focused on the specific role of CDK1 in pancreatic cancer cells and its CSC subpopulation. CDK1 expression was relatively high in PDAC and PCSCs, and its upregulation was correlated with poor prognosis. Suppression of CDK1 by fisetin reduced tumor stemness and phosphorylation of CDK1 and STAT3. The acetylation of CDK1 at lysine 33 weakened its phosphorylation, thus playing an important role in phosphorylated kinase function. The reduction in CDK1 expression and the impairment of its kinase function affected STAT3 phosphorylation, which was reported as a critical molecule for maintaining CSC subpopulations. Additionally, fisetin enhanced the effect of gemcitabine in vivo and in vitro through regulating post-translational modifications (PTMs) of CDK1–STAT3 signaling. Our results highlighted the role of post-translational modification of CDK1–STAT3 signaling in maintaining cancer stemness of PDAC and suggested that inhibition of the CDK1–STAT3 axis by fisetin helps to suppress PDAC cell proliferation through eliminating PCSC subsets.

## Results

### CDK1 played an important role in the cancer stem cell properties of human pancreatic cancer

**Fig. 1:**
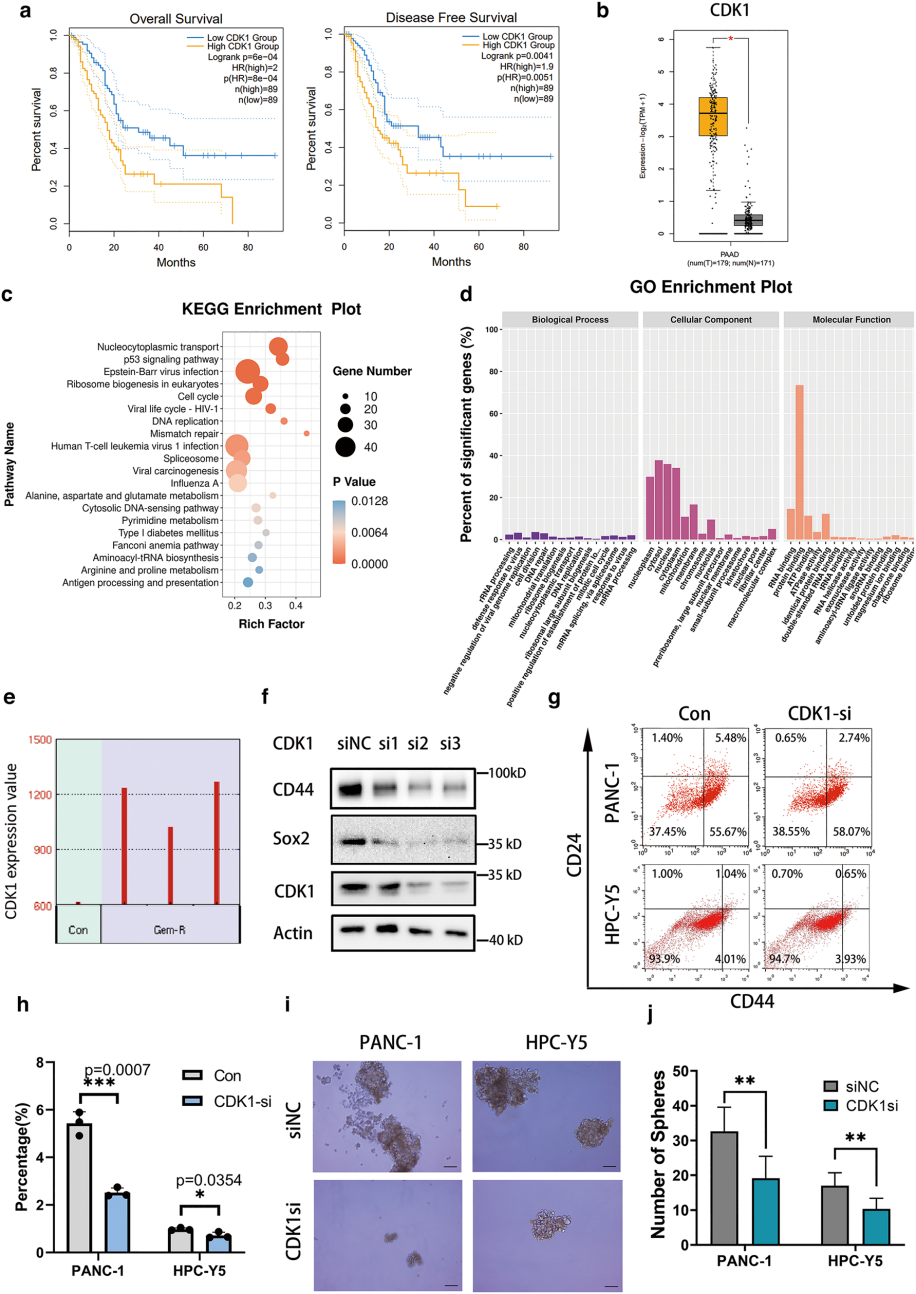
CDK1 played an important role in cancer stem cell properties in PDAC cells. **a** The overall and disease free survival analysis of patients with PDAC from GEPIA database (http://gepia.cancer-pku.cn/). Patients with low levels of CDK1 have a better prognosis. Data are presented as mean ± SD (n = 89). **b** Expression of CDK1 was higher in pancreatic cancer than in tumor-adjacent tissue. T, tumor; N, tumor-adjacent tissue. *P < 0.05. **c**, **d** KEGG and GO enrichment analyses of differential genes in gemcitabine-resistant pancreatic cancer PANC-1 cells (GSE80617). **e** Relative mRNA expression of CDK1 in normal and gemcitabine-resistant pancreatic cancer PANC-1 cells (GSE80617). **f** Western blot analysis showed that the expression of pancreatic CSC markers CD44 and Sox2 were significantly decreased after CDK1 silencing in pancreatic cancer cell lines. siNC: negative control; si1-3: silencing 1–3. **g** Representative flow cytometry plots for CD44 and CD24 expression in pancreatic cancer PANC-1 and HPC-Y5 cells with CDK1 silencing. **h** Statistical plot of ratio of CD44+/CD24+ positive cells in control or CDK1 silencing PANC-1 and HPC-Y5 cells. Data are presented as mean ± SD (n = 3); *P < 0.05. Con: Control, CDK1si: CDK1 silencing. **i**, **j** Sphere formation assay was used to determine the effect of CDK1 silencing for pancreatic cancer stemness. Scale bars, 100 μm. Data are presented as mean ± SD (n = 6). **P < 0.01

Correlation analysis using PDAC data from GEPIA database demonstrated that CDK1 expression in PDAC tumor tissue was higher than that in non-tumor tissue, while high levels of CDK1 were associated with poor overall survival or disease-free survival (Fig. 1a, b). KEGG and GO enrichment analysis revealed that cell cycle signaling pathway was significantly different in gemcitabine-resistant human pancreatic cancer PANC-1 cells, among which CDK1 was highly expressed in drug-resistant cells (Fig. 1c–e).

Based on previous investigations highlighting the significance of CDK1 in maintaining stem cell pluripotency [10–12], we hypothesized that CDK1 was related to PCSC function in pancreatic cancer. Here, we demonstrated that silencing *CDK1* reduced CD44 and Sox2 expression, which suggested that CDK1 might influence stem cell pluripotency in PDAC (Fig. 1f). Simultaneously, knockdown of *CDK1* reduced a subset of CSCs and impaired sphere formation of PANC-1 and HPC-Y5 cells, suggesting that CDK1 plays an important role in PCSC stemness (Fig. 1g–j).

### Inhibition of CDK1 by fisetin impaired the cancer stem cell properties of human pancreatic cancer cells

**Fig. 2:**
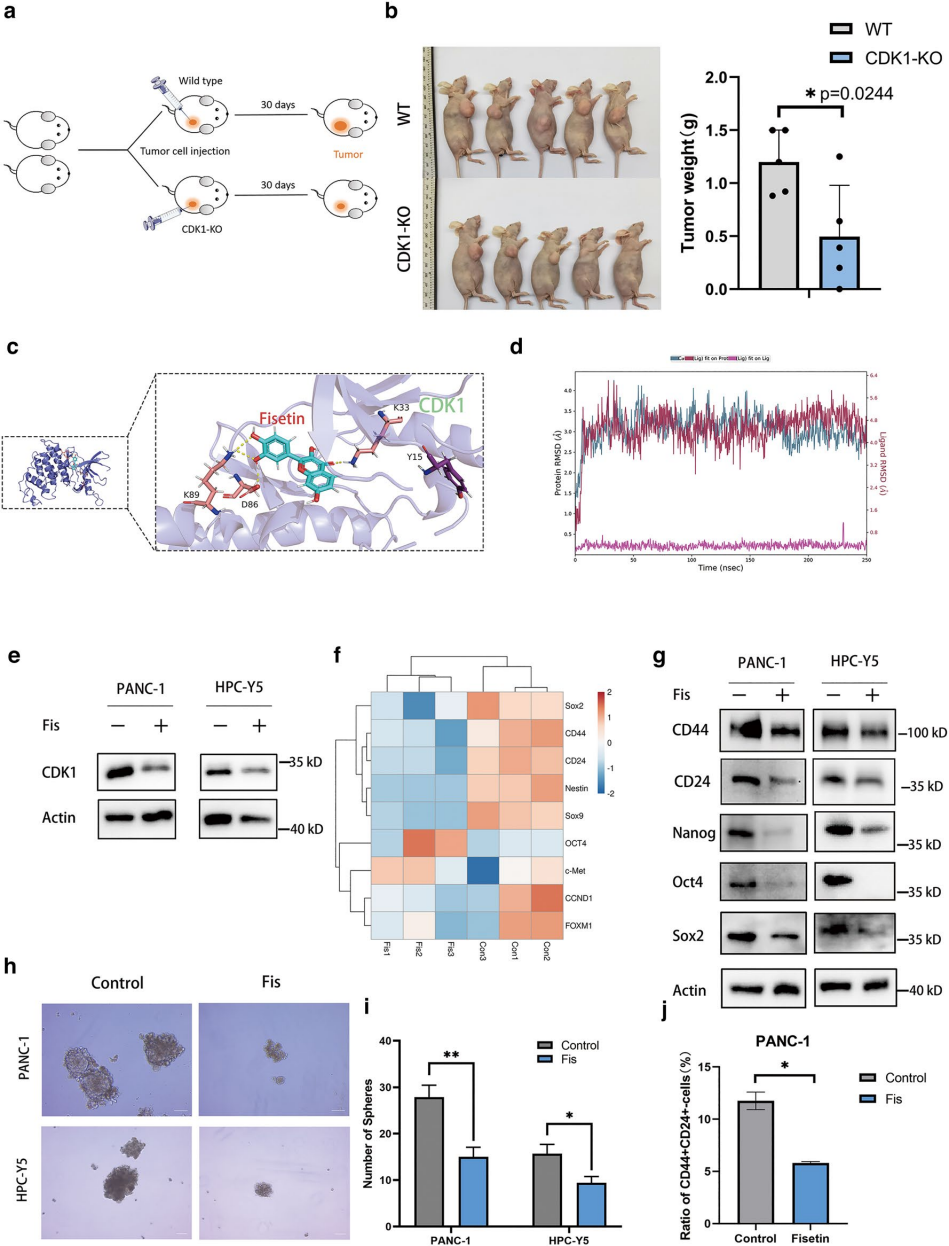
CDK1 and cancer stemness related genes were inhibited by fisetin.** a** Flow chart of nude mice model of CDK1 silencing PANC-1 cells. CDK1-KO: CDK1-knockout. **b** Photograph and weights of xenograft tumors of wild type (WT) and CDK1-Knockout (KO) mice at day 30 after tumor cells injection. Data are presented as mean ± SD (n = 5); *P < 0.05. **c** Three-dimensional illustration of docked CDK1–fisetin complex. CDK1 is represented as slate cartoon. Fisetin is showed as cyan sticks, binding sites are displayed a series of pink sticks and hydrogen bonds are represented as yellow dashes. **d** RMSD plot of CDK1-Fisetin. Cα-RMSD, ligand RMSD of ligand fit on protein or ligand fit on ligand are represented as deep blue, deep red and pink lines, respectively. **e** Western blot analysis showed that expression of CDK1 were significantly inhibited by fisetin in both pancreatic cancer PANC-1 and HPC-Y5 cells. **f** Heat map representing mRNA expression levels of stemness related genes in RNA-sequencing of PANC-1 cells. Each column indicates one sample and each row represents one mRNA. Orange and blue indicate high and low expression, respectively. **g** Western blot analysis was used to determine the protein expressions of Nanog, Oct4 and Sox2 with treatment of fisetin for 48 h. **h**, **i** Sphere formation assay of PDAC cells. After 100 μM fisetin treatment for 48 h, PANC-1 and HPC-Y5 cells were subjected to the tumor sphere-formation assay in ultra-low cluster plates. Scale bars, 100 μm. Data are presented as mean ± SD (n = 3),*P < 0.05, **P < 0.01. **j** Statistical plot of ratio of CD44 + /CD24 + positive cells in control or fisetin treatment PANC-1 cell line. Data are presented as mean ± SD (n = 3); *P < 0.05

To further determine the role of CDK1 in pancreatic cancer in vivo, we constructed wild type and CDK1-silenced nude mice xenograft models (Fig. 2a, Additional file 1: Figure S10a). The results showed that knockdown of CDK1 obviously suppressed tumor growth in this mouse model (Fig. 2b). Our previous studies demonstrated that fisetin can inhibit the proliferation of pancreatic cancer cells [15]. Via Prewizard modification and PyMol 2.2.0 viaualization (Fig. 2c), we found out all functional residues applied to form conventional hydrogen bond, there are K33, D86,and K89. With assistance of these residues, the binding energy of two parts achieved − 8.5 kcal/mol. Accordingly, the result was meant to be pretty. Futhermore, MD simulation were used to assess the stability and specificity of fisetin binding psoe. Based on the RMSD plot (Fig. 2d), it can be identifed that the mode of binding between Fisetin and CDK1 could be allosteric in nature, since conformational changes were induced within the protein. Western blotting further demonstrated that fisetin significantly reduced CDK1 expression (Fig. 2e).

RNA sequencing revealed the expression of PCSC markers and pluripotency-associated genes such as *SOX2*, *NES*, *SOX9*, *NANOG*, and *OCT4*. Expression of these pluripotency-associated genes was significantly lower in PANC-1 cells following treatment with fisetin (Fig. 2f). The expression levels of CD44, CD24, Sox2, OCT4, and NANOG for PDAC cells were further validated by western blotting (Fig. 2g). Sphere formation assay suggested that fisetin suppressed sphere size and numbers in both PANC-1 and HPC-Y5 cells (Fig. 2h, i). Here, we further explored whether fisetin functions in regulating PCSC subgroups. PCSC biomarker expression and sphere formation assays are often used to analyze PCSCs [18, 19]. As previously reported, cells were phenotyped by flow cytometry for the expression of pancreatic CSC markers CD44 and CD24. The results showed that fisetin reduced the ratio of CD44+/CD24+ cells and increased that of CD44−/CD24− cells in human pancreatic cancer PANC1 cells (Fig. 2j). As for pancreatic cancer HPC-Y5 cells, although the ratio of CD44+/CD24+ cells did not exhibit a significant change due to the small size of the double-positive cell subset, the subpopulation of CD44−/CD24− cells was increased following fisetin treatment (Additional file 1: Figure S1a,b). These results suggested that fisetin suppressed CSC properties in PDAC.

To further study the function of fisetin, we identified potential changes in proteins after fisetin treatment for PANC-1 cells through a stable isotope labeling by amino acids in a cell culture (SILAC)-based quantitative proteomic approach. A total of 5136 proteins were identified, among which 4071 proteins contained quantitative information (Additional file 2: Table S1). Among these quantified proteins, 1346 were changed more than 1.2-fold (566 upregulated and 273 downregulated; P < 0.05). All differentially expressed proteins were divided into four quantiles (Q1–Q4) based on their ratios of fold change: Q1 (> 0 ratio ≤ 1/1.5), Q2 (> 1/1.5 ratio ≤ 1/1.2), Q3 (> 1.2 ratio ≤ 1.5), and Q4 (ratio > 1.5) (Additional file 1: Figure S1c). GO classification, KEGG, and protein domain enrichment were performed for each Q group, and cluster analysis was conducted to identify the correlation of protein functions with different expression multiples (Additional file 1: Figure S1d, S2, S3, S4a, b). The results showed that fisetin influenced several pathways including PI3K–AKT, NF-κB, and ECM–receptor signaling, which have been reported previously in other studies (Additional file 1: Figure S5a). In addition, domain functional enrichment analysis identified that fisetin suppressed proteins with EGF-like domains, protein kinase domains, and the C-terminal domain of protein kinase, which indicated that fisetin inhibited protein kinase-related signaling in PANC-1 cells (Additional file 1: Figure S5b, c). Moreover, 14 protein kinase-like domain-associated genes including *CDK1*, *AKT2*, *PTK2*, and *PTK7* were reduced by fisetin (Additional file 3: Table S2).

### Protein phosphorylation array revealed reduced phosphorylation of CDK1 and STAT3

**Fig. 3:**
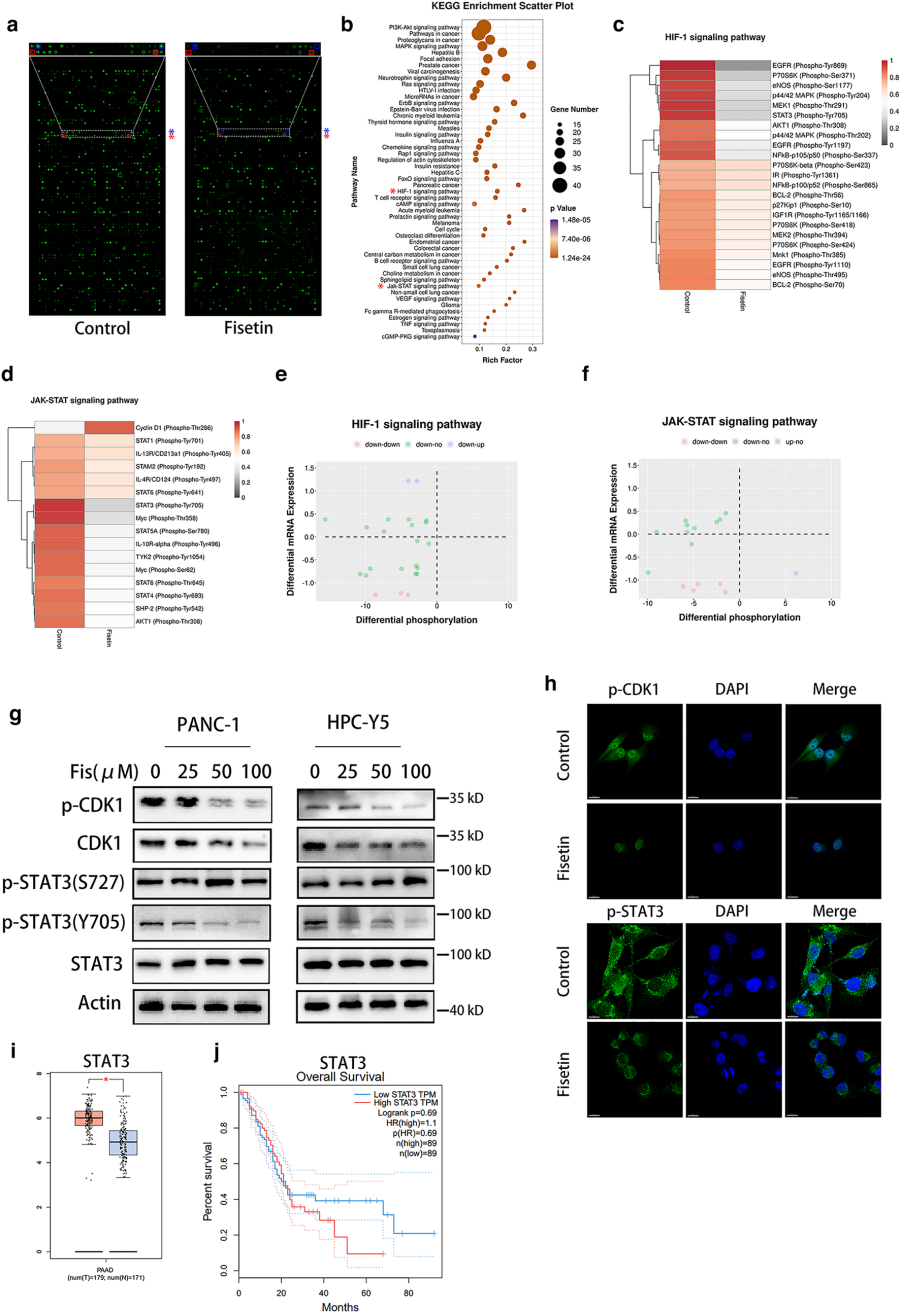
Phosphorylations of CDK1 and STAT3 were reduced by fisetin. **a** Phospho-array analysis of PANC-1 cells treated with fisetin. *Blue and red squares represented phosphorylation of STAT3-Y705 and CDK1-Y15, respectively. **b** KEGG pathway enrichment of proteins with differential levels of phosphorylation after fisetin treatment. Red asterisk (*) indicated that CSC related pathways as HIF-1 signaling and JAK–STAT signaling were enriched by KEGG pathway analysis. **c**, **d** Heat map representing phosphorylation of proteins enriched in HIF-1 signaling and JAK–STAT signaling pathways (data are presented as mean, n = 2). **e**, **f** Integrated transcriptome and phospho-array analysis of HIF-1 signaling and JAK–STAT signaling pathways in PANC-1 cells treated with fisetin. Red, green and blue spots indicated that both mRNA and phosphorylation of proteins decreased (mRNA: FC ≥ 2, phosphorylation: FC ≥ 1.12, P < 0.05), only phosphorylation of proteins reduced, mRNA increased and phosphorylation of proteins decreased, respectively. **g** Western blot analysis was used to test the phosphorylation of CDK1 and STAT3 in PDAC cells treated by fisetin. **h** Expressions of p-CDK1 (Y15) and p-STAT3 (Y705) in control and fisetin treatemt groups were examined by immunofluorescence. Scale Bars 15 μm. **i**, **j** The survival analysis of patients with PDAC from GEPIA database. STAT3 is highly expressed in pancreatic cancer tissue. High levels of STAT3 are associated with poor outcomes. Data are presented as mean ± SD (n = 89). T, tumor; N, tumor-adjacent tissue. *P < 0.05

Because CDK1 is a classical phosphorylated kinase, we revealed the dynamic regulation of multiple pathways in response to fisetin treatment via comprehensive protein phosphorylation array, in which the phosphorylation intensity of 584 proteins could be measured. Phosphorylation array revealed changes in the phosphorylation of 166 proteins, in which the phosphorylation of CDK1 at tyrosine 15 and STAT3 at tyrosine 705 were decreased (Fig. 3a). KEGG enrichment of differentially phosphorylated genes indicated that several pathways including the JAK–STAT, HIF-1, PI3K–AKT, and MAPK signaling cascades, which play critical roles in tumor proliferation, were regulated by fisetin in PDAC (Fig. 3b). Heatmaps of differentially phosphorylated proteins in the HIF-1 and JAK–STAT signaling pathways showed that phosphorylation at 23 sites of 14 proteins in HIF-1 signaling and at 15 sites of 13 proteins in JAK–STAT signaling was downregulated by fisetin (Fig. 3c, d). Integrated transcriptome and protein phosphorylation array indicated that 11 proteins in HIF-1 signaling and 9 proteins in JAK–STAT signaling had merely reduced phosphorylation, while mRNA and total protein levels did not change, suggesting that the function of these proteins rely on the regulation of post-translational modification (Fig. 3e, f, Additional file 4: Table S3, Additional file 5: Table S4). Combining differentially phosphorylated proteins from the HIF-1 and JAK–STAT pathways revealed that phosphorylation of STAT3 at Y705 functioned in both pathways. The reduction in CDK1-Y15 and STAT3-Y705 phosphorylation was validated by western blotting and immunofluorescence (Fig. 3g, h). Since both of the CDK1 and p-CDK1-Y15 were down regulated, we quantificated the relative fold change of expression of them by image J. The results revealed that relative expression of p-CDk1-Y15 was significantly reduced by fisetin (Additional file 1: Figure S10b). To understand the reduction of CDK1 by fisetin, we detected the CDK1 mRNA levels in PDAC cell lines. The results showed that fisetin did not reduce the mRNA of CDK1 significantly (Additional file 1: Figure S10c). Then we used proteasome inhibitor MG132 to investigate whether fisetin promote CDK1 degradation through post-translational modifications. The results revealed that MG132 restored the expression of CDK1 in pancreatic cancer cells with treatment of fisetin (Additional file 1: Figure S10d). Moreover, the immunoprecipitation further demonstrated that fisetin obviously induce the ubiquitination of CDK1, which will result in degradation through proteasome (Additional file 1: Figure S10e).

TCGA data indicated that STAT3 levels in PDAC were higher than in tumor-adjacent tissue, which was related to the poor prognosis of PDAC patients (Fig. 3i, j). Recent studies have found that CDK1 activates JAK–STAT3 signaling in lung and colorectal cancer [20, 21]. Furthermore, we demonstrated that both CDK1 and STAT3 were highly expressed in tumor spheres from PANC-1 cells (Additional file 1: Figure S7a). Thus, we speculated that CDK1 might activate STAT3 via its kinase function to influence the maintenance of cancer stemness in pancreatic cancer.

### Lysine acetylation proteomics identified acetylation of CDK1

**Fig. 4:**
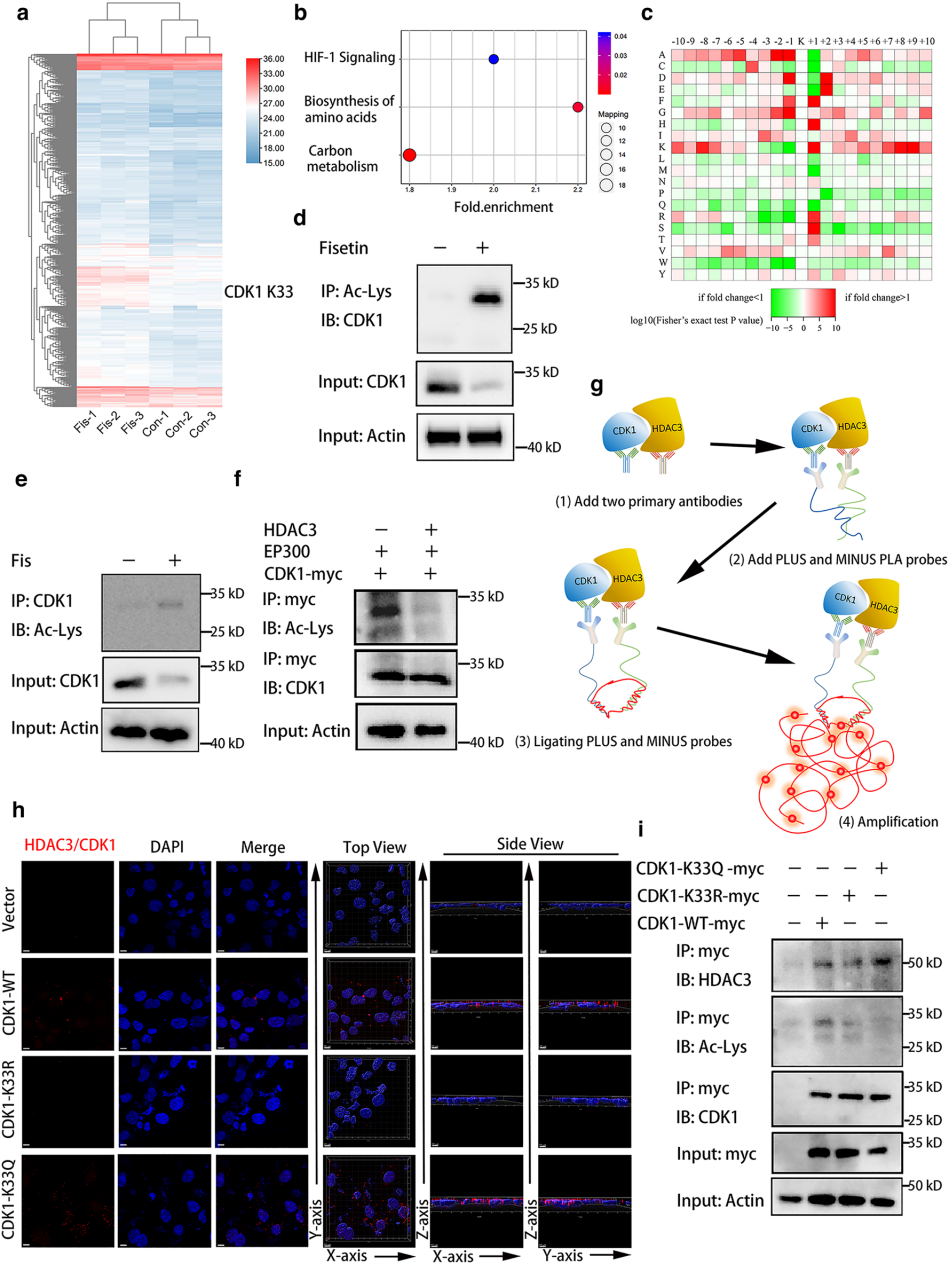
Acetylation of CDK1 at K33 was regulated by HDAC3. **a** Heat map representing acetylation levels of proteins identified by acetyl-proteomics in PANC-1 cells after fisetin treatment. Red and blue indicated high and low acetylation levels, respectively. **b** KEGG pathway enrichment of proteins with differential expression acetylation after above treatment. **c** Heat maps of motif enrichment of amino acids upstream and downstream of all identified acetylated modification sites. Red and green represented that the amino acid was significantly enriched or reduced near the modification site, respectively. **d**, **e** Co-immunoprecipitation was used to detect the acetylation of CDK1 with fisetin treatment; Ac-Lys, Acetyl Lysine. **f** Immunoprecipitation was used to test the the deacetylation effect of HDAC3 to CDK1. **g**, **h** In situ proximity ligation assay (PLA) was used to determine the location and interaction between HDAC3 with CDK1-wild type and mutants. Top and side views of reconstructed 3D images of confocal micrographs were generated by Imaris software. Red spots represented positive interaction between HDAC3 and CDK1. Scale bars 10 μm. **i** Immunoprecipitation indicated that HDAC3 decreased the acetylation of CDK1 K33, and acetylation mimics of CDK1 K33 had highly binding capacity with HDAC3, while CDK1-K33R mutant impaired the interaction with HDAC3

PTMs such as acetylation and phosphorylation exert diverse effects on multiple cancer signaling pathways. To further understand the mechanism of fisetin and its effect on CDK1 in PDAC, we performed quantitative profiling of acetylation sites via lysine acetylation (Ac-Lys) proteomics in PANC-1 cells after fisetin treatment. A total of 3619 acetylation sites were identified on 1402 proteins, among which 3189 sites on 1317 proteins had quantitative information (Additional file 1: Figure S5a). After normalization of proteome quantitative data, the effect of expression abundance on modification was removed, and Ac-Lys proteomics quantified 2800 acetylation sites in 1039 proteins in PANC-1 cells (Fig. 4a). Among these acetylated lysine sites, 368 were changed by more than 1.2-fold (P < 0.05) including 307 that were upregulated and 61 that were downregulated (Additional file 1: Figure S5b). We identified that acetylation of CDK1 at lysine 33 (K33) site was increased by fisetin treatment. GO enrichment of differential acetylated proteins showed that acetylation of pathways including protein folding, protein localization involved in protein folding, unfolded protein binding, and ATP binding were upregulated by fisetin, indicating that acetylation induced by fisetin played an important role in protein folding in PDAC cells (Additional file 1: Figure S5c). Furthermore, KEGG enrichment revealed that acetylation levels of HIF-1 signaling, carbon metabolism, and biosynthesis of amino acids pathways were upregulated by fisetin (Fig. 4b). Motif analysis demonstrated that 19 types of conserved motifs that were prominently enriched at different levels of abundance and acetylation of CDK1-K33 were related to motif ..........KK........., with the .........AKK......... motif exhibiting the highest motif score (Additional file 1: Figure S6, Fig. 4c). We further confirmed that the acetylation of CDK1 was upregulated by fisetin through coimmunoprecipitation experiments (Fig. 4d, e). To clarify the acetylase of CDK1, we performed immunoprecipitation studies and determined that EP300 was the major acetylase of CDK1 (Additional file 1: Figure S5d). A previous study reported that histone deacetylase 3 (HDAC3) regulated CDK1 levels through post-translational regulation [22]; thus, we supposed that HDAC3 might control the acetylation level of CDK1 in PDAC cells. Immunoprecipitation proved that HDAC3 overexpression inhibited the acetylation of CDK1 (Fig. 4f).

To determine whether acetylation of CDK1 regulated by HDAC3 was at K33, we constructed a K33 mutant plasmid, CDK1-K33R, and a mutant CDK1-K33Q in which K33 was substituted by glutamine to mimic acetylation. We next examined the localization and interaction between HDAC3 and CDK1 in PANC-1 cells using an in situ proximity ligation assay (PLA). PLA revealed that CDK1 physically interacted with HDAC3 (the distance between the two proteins was less than 40 nm), indicating that HDAC3 and CDK1 are likely to reside in the same protein complex, while CDK1-K33R mutation impaired the interaction and CDK1-K33Q increased the binding capacity with HDAC3 (Fig. 4g, h). The findings were further confirmed by immunoprecipitation of HDAC3 and CDK1 mutants, suggesting that HDAC3 might be attracted by acetylation of CDK1 at K33 (Fig. 4i). Together, these results suggested that acetylation of CDK1 at K33 was upregulated after fisetin treatment, while HDAC3 could reduce the acetylation of CDK1 at K33.

### CDK1 regulated cancer stemness properties through activating STAT3

**Fig. 5:**
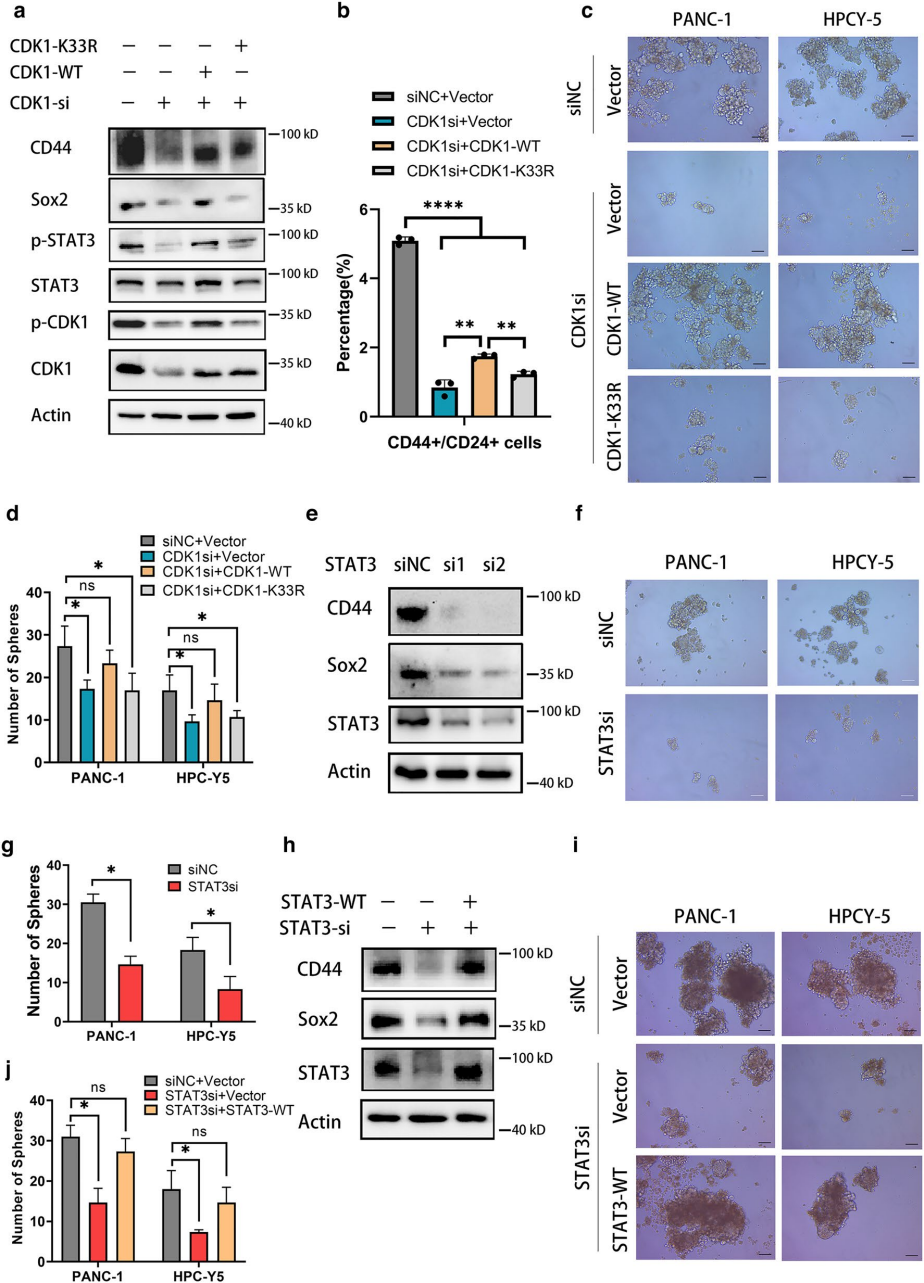
CDK1 regulated cancer stemness through phosphorylating STAT3. **a** Silencing CDK1 reduced phosphorylation of STAT3 Y705. CDK1-WT and CDK1-K33R was overexpressed in CDK1 silencing PANC-1 cells. Western blot analysis showed that silencing CDK1 decreased levels fo CD44, Sox2 and p-STAT3 (Y705), which can be rescued by over-expressing CDK1-WT not its mutant. **b** Flow cytometry showed that over-expressing CDK1-WT not K33R mutant rescued decreasing of CD44+/CD24+ cancer stem cells in PDAC. Data are presented as mean ± SD (n = 3) *P < 0.05. **c, d** Sphere formation assay showed that over-expressing CDK1-WT not K33R mutant contributed to tumor stemness in PDAC cells. Data are from three independent experiments. Scale bars, 100 μm. *P < 0.05. **e** Expressions of CD44 and Sox2 in STAT3 silencing PANC-1 cells were examined by western blot. **f, g** Silencing STAT3 inhibited tumor stemness in PDAC cells. Sphere formation assay was used to detect cancer stemness in STAT3 silencing PDAC cells. Data are presented as mean ± SD (n = 3). Scale bars, 100 μm. *P < 0.05. **h** Western blot analysis showed that silencing STAT3 reduced levels fo CD44 and Sox2, which can be rescued by over-expressing STAT3 wild type. **i, j** STAT3 contributed to maintaining of tumor stemness in PDAC cells. Sphere formation assay indicated that over-expressing STAT3 enhanced sphere forming capacity which was impaired by silencing STAT3 in PDAC cells. Data are from three independent experiments. Scale bar, 100 μm. *P < 0.05

The process of regulation by CDK1 and the role of acetylation of CDK1-K33 in JAK–STAT3 signaling is still unknown. To elucidate the mechanism of CDK1 in JAK–STAT3 signaling, we conducted rescue assays to demonstrate the effect of CDK1 on STAT3. The results showed that *CDK1* knockdown reduced the expression of CD44 and Sox2 as well as the phosphorylation of STAT3-Y705, and overexpression of CDK1-wildtype (CDK1-WT) but not its mutant partially rescued this phenomenon (Fig. 5a). Flow cytometry and sphere formation assays further validated the function of the conserved catalytic residue K33 of CDK1 in maintaining the stemness of PDAC cells (Fig. 5b–d). Knockdown of *STAT3* also reduced the expression of CD44 and Sox2 and impaired the sphere formation capacity of PDAC cells (Fig. 5e–g). These findings caused by knockdown of *STAT3* could be reversed by overexpressing STAT3-WT, suggesting that STAT3 maintained the PDAC stemness characteristics (Fig. 5h–j). Moreover, the inhibition of CD44 and Sox2 by silencing of *CDK1* could be partially rescued by overexpressing STAT3-WT, indicating that CDK1 regulated the expression of CD44 and Sox2 through STAT3 (Additional file 1: Figure S7b). These results demonstrated that CDK1 regulated cancer stemness properties through activating STAT3 signaling. Meanwhile, we purified the cancer stem cells via multiple generation of sphere formatting. We found that CDK1, p-CDK1 Y15, p-STAT3 Y705, CD44 and Sox2 were significantly diminished by fisetin in purified pancreatic oncospheres (Additional file 1: Figure S7c). Moreover, fisetin showed direct function in reducing growth and sphere formation capacity of purified pancreatic oncospheres (Additional file 1: Figure S7d-e). Together, these results showed that inhibiting CDK1–STAT3 pathway by fisetin significantly impaired the stemness of purified PCSCs.

### STAT3 was interacted and activated by CDK1

**Fig. 6:**
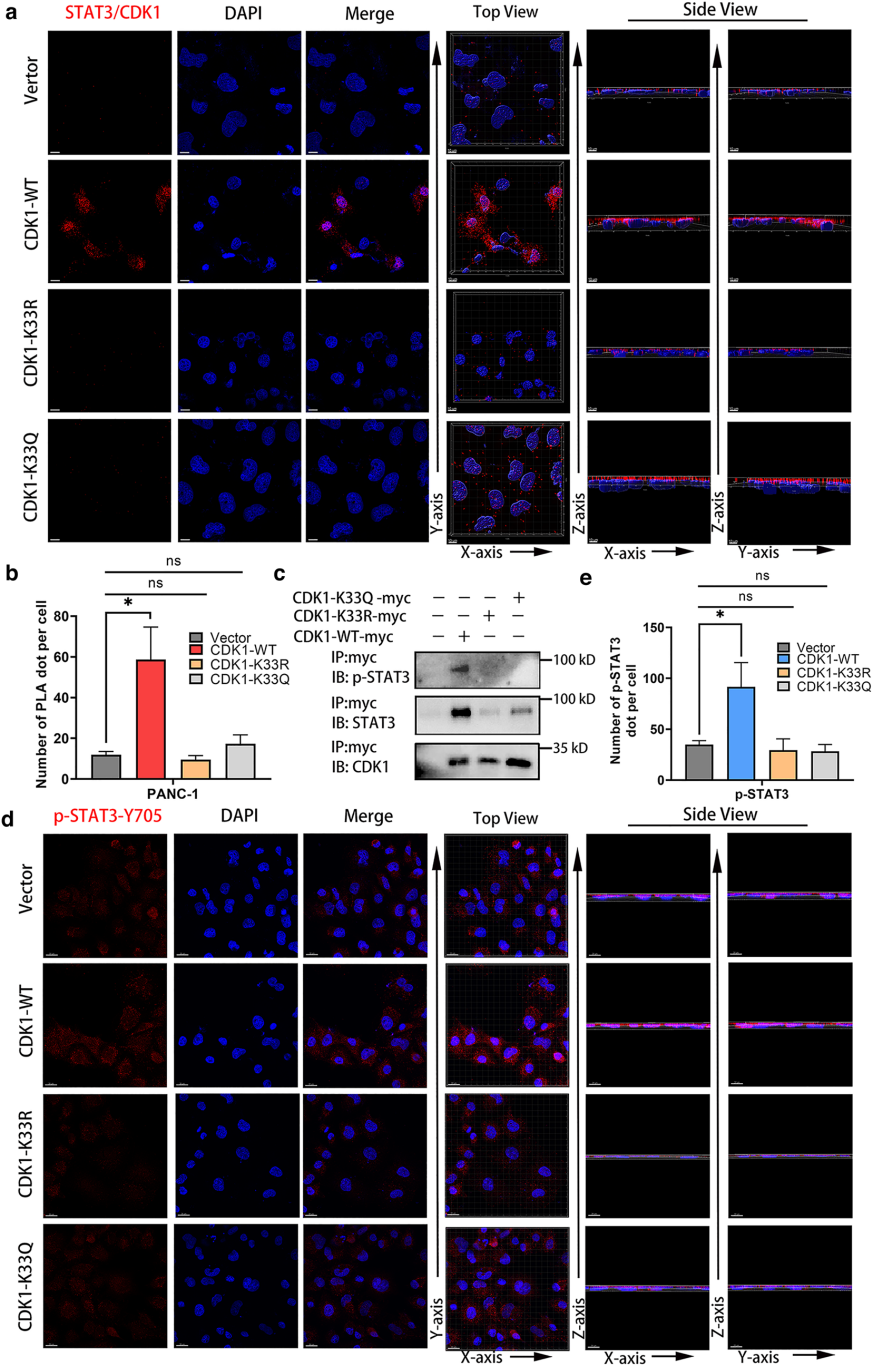
STAT3 was interacted and activated by CDK1. **a**, **b** The direct interaction and location between STAT3 with CDK1-wild type and mutants were examined by in situ PLA analysis. Top and side views of reconstructed 3D images of confocal micrographs were generated by Imaris software. Red spots represented positive interaction between STAT3 and CDK1. Scale bars 10 μm. Number of positive spots was counted by Imaris software, results were from three independent experiments. *P < 0.05, ns, no significance. **c** CDK1 interacted with STAT3. Myc-tagged CDK1-WT or K33R/Q mutants were over-expressed in PANC-1 cells. The interaction between CDK1 and STAT3 was determined by immunoprecipitation and western blot analysis. CDK1-WT interacted with STAT3 while CDK1 K33R/Q mutants weakened binding capacity with STAT3 and p-STAT3 (Y705). **d**, **e** Immunofluorescence was used to detect the phosphorylation of STAT3 Y705. CDK1-WT not K33R/Q mutants increased levels of p-STAT3 Y705. Scale bars 20 μm. The spots of p-STAT3 were counted by Imaris software, results were from three independent experiments. *P < 0.05, ns, no significance

PLA demonstrated that CDK1 physically interacted with STAT3 in both the nucleus and cytoplasm, while CDK1-K33R and K33Q mutation impaired this interaction, which suggested that the conserved catalytic residue K33 was important for forming a complex with STAT3 (Fig. 6a, b). The interaction between CDK1 and STAT3 was further examined by immunoprecipitation and western blot analyses. CDK1-WT interacted with STAT3 and p-STAT3, while CDK1 K33R/Q mutants had weakened binding capacity with STAT3 and p-STAT3, suggesting that mutation or mimicking acetylation of CDK1 K33 impaired the phosphorylation of STAT3 (Fig. 6c). Immunofluorescence also indicated that mutation or mimicking acetylation of CDK1 K33 impaired the phosphorylation of STAT3 Y705 in both the nucleus and cytoplasm, which further illustrated the function of K33 residue on CDK1 (Fig. 6d, e).

### HDAC3 regulated STAT3 phosphorylation

Because HDAC3 has been reported to regulate the phosphorylation of STAT3 Y705 in other cancer types [23, 24], we speculated that HDAC3 might influence the phosphorylation of STAT3 Y705 via activation of CDK1. The results showed that *HDAC3* silencing reduced the phosphorylation of CDK1 Y15 and STAT3 Y705 followed by reduction of CD44 and Sox2, which resulted in the impairment of cancer cell stemness (Additional file 1: Figure S8a–c). Conversely, HDAC3 overexpression enhanced the expression of CD44 and Sox2 and the phosphorylation of CDK1 and STAT3, which in turn elevated the stemness of PDAC cells (Additional file 1: Figure S8d–f). Interestingly, fisetin also inhibited HDAC3 expression, suggesting that fisetin not only boycotted CDK1 expression directly, but inhibited PTMs of CDK1 via HDAC3 (Additional file 1: Figure S8g).

### Fisetin synergistically potentiated the anti-tumor effects of gemcitabine in vivo and in vitro

**Fig. 7:**
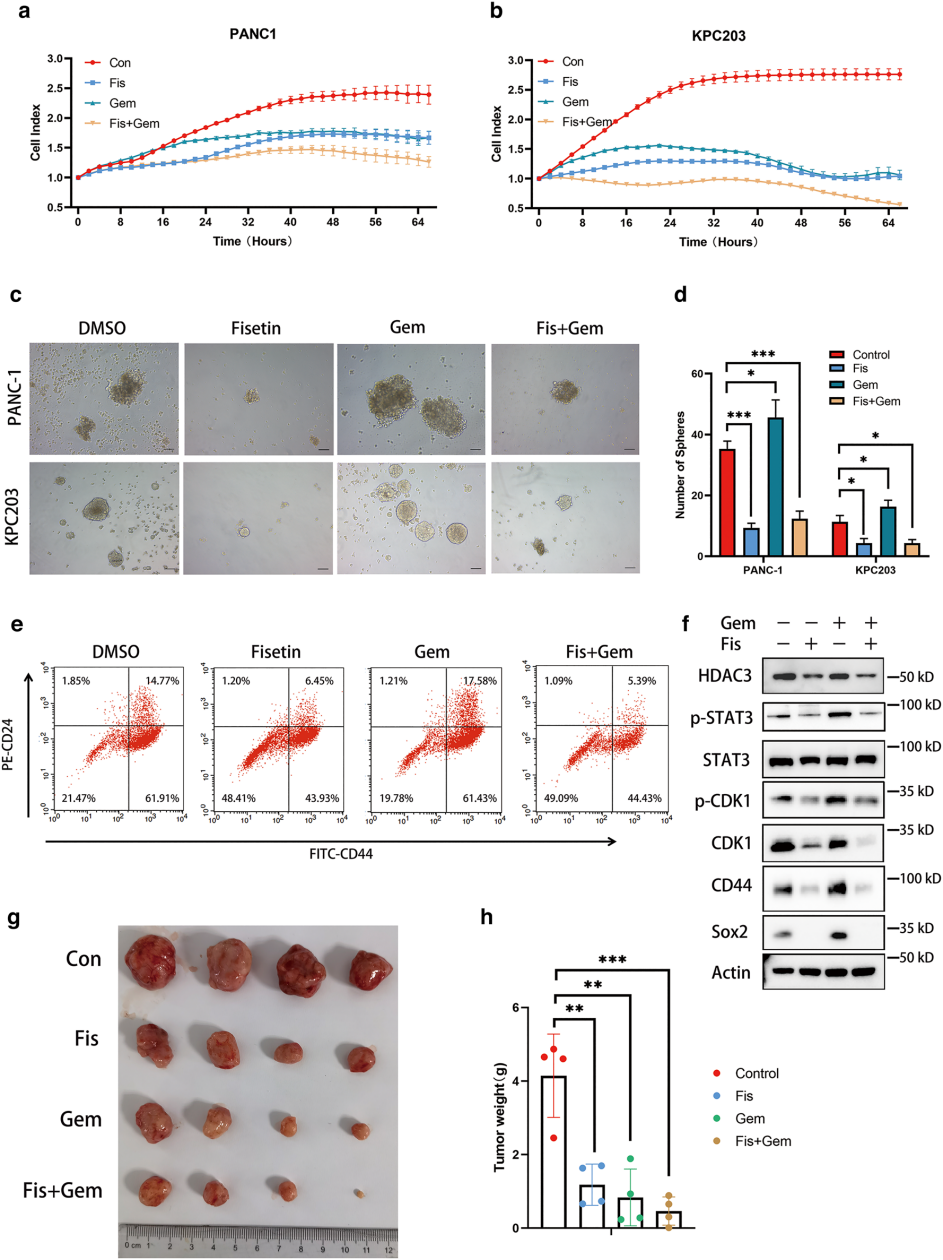
Inhibiting CDK1-STAT3 signaling by fisetin enhanced effect of gemcitabine through suppressing cancer stemness. **a**, **b** Real-time proliferation analysis of human PANC-1 cells and mice KPC203 cells. Cells were treated with DMSO, fisetin (100 µM), gemcitabine (10 µM) or combination therapy of fisetin (100 µM) and gemcitabine (10 µM) for three days. Cell growth was monitored using the Incucyte SX5 system, as area of cells was recorded every hour. The times on the x- axis indicated the times after treatment. The y- axis reflected phase area of cells per well normalized to 0 h. (n = 6, mean ± SD). Fis, fisetin, Gem, gemcitabine. **c**, **d** Sphere formation assay of human PANC-1 cells and mice KPC203 cells with DMSO, fisetin alone, gemcitabine alone or combination therapy. Cells were pretreated with fisetin, gemcitabine or combination therapy for 48 h, then 1 × 10^3^ cells were seed in ultra low ultra-low cluster plates to perform sphere forming analysis. Scale bars 100 μm. Data are presented as mean ± SD (n = 3). *P < 0.05, ***P < 0.001. **e** Representative flow cytometry plots for CD44 and CD24 expression in human pancreatic cancer PANC-1 cells with DMSO, fisetin alone, gemcitabine alone or combination therapy treatment as above. Proportion of CD44+/CD24+ positive cells were increased by gemcitabine, which can be attenuated by fisetin in combination group. **f** Protein levels of HDAC3, p-STAT3, STAT3, p-CDK1, CDK1, CD44 and Sox2 in PANC-1 cells treated with the indicated treatment, was detected by western blot. **g** Photographs and **h** weights of allograft tumors of KPC (Kras^LSL−G12D^ p53^LSL−R172H^ Pdx-1-Cre) mice at day 21 after first treatment. Data are presented as mean ± SD (n = 4); **P < 0.01, ***P < 0.001

Because PDAC acquires resistance through the enhanced stemness of cancer cells following gemcitabine treatment [7, 16, 17], we supposed that fisetin might enhance the effect of gemcitabine through inhibition of PDAC cell stemness. The drug combination index (CI) of fisetin and gemcitabine was calculated using CompuSyn software, with CI < 1 representing synergy (Additional file 1: Figure S9a). We found that 100 µM fisetin combined with 10 µM gemcitabine showed a preferable CI, and therefore we tested a fisetin–gemcitabine combination regimen for PANC-1 cell real-time killing measured by IncuCyte live-cell analysis (Fig. 7a). A 3-day single agent treatment with 100 µM fisetin or 10 µM gemcitabine induced a similar degree of cell death, while the combination regimen showed better anti-proliferation function; this was also observed in KPC203 cells generated from C57B1/6 KPC (*Kras*^*LSL−G12D*^; *p53*^*LSL−R172H*^; *Pdx-1-Cre*) mice (Fig. 7a, b). Sphere formation assay revealed that fisetin reduced the cancer stemness promoted by gemcitabine in human PANC-1 cells and KPC mice KPC203 cells, as well as the proportion of CD44+/CD24+ cells among PANC-1 cells, further proving the results (Fig. 7c–e). The expression of p-CDK1, p-STAT3, CD44, and Sox2 was increased by gemcitabine, while fisetin could reverse the upregulation of these proteins in the combination therapy group, suggesting that inhibition of cancer stemness-related proteins by fisetin enhanced the effects of gemcitabine (Fig. 7f).

**Fig. 8:**
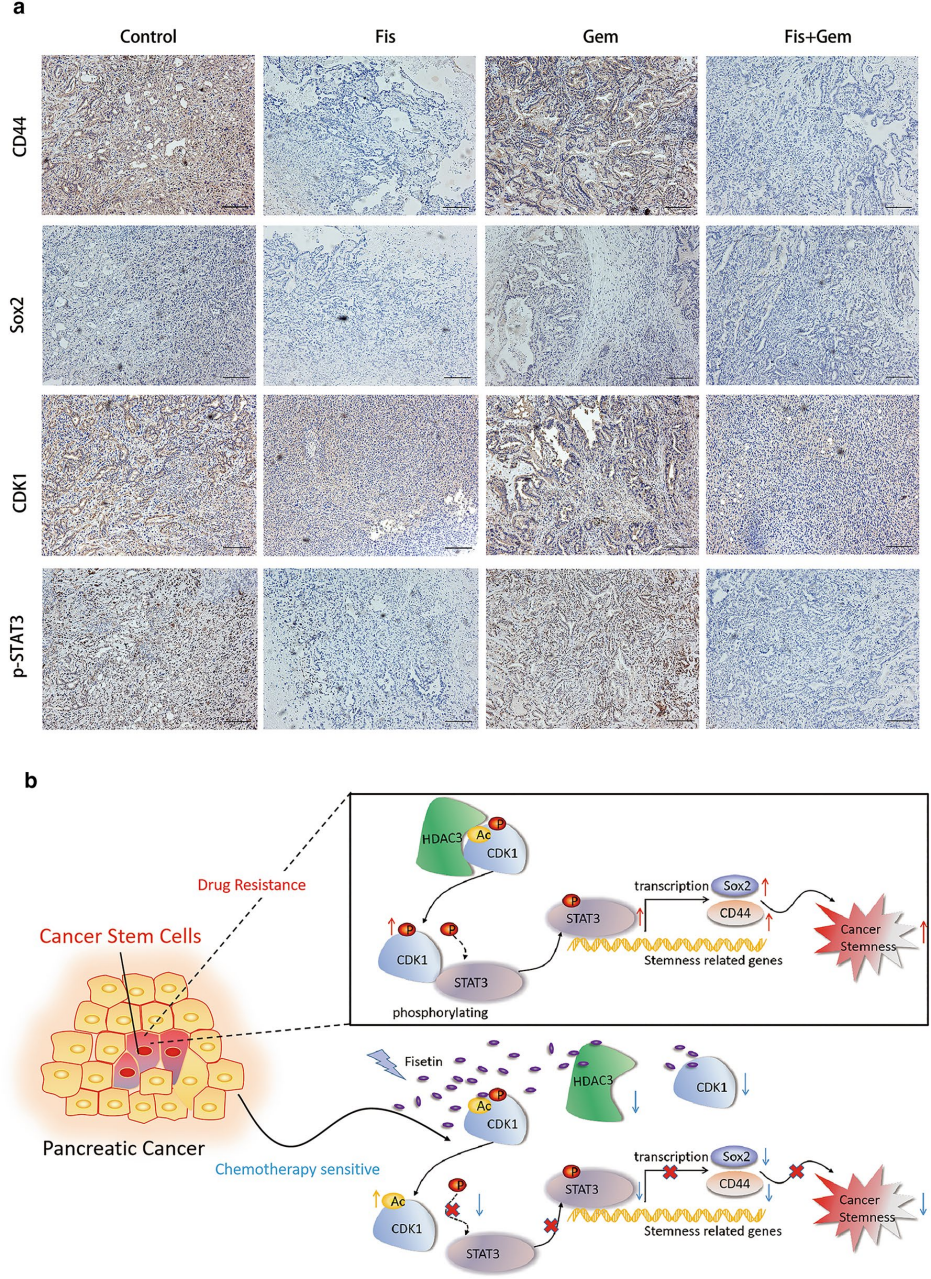
Fisetin inhibited CDK1-STAT3 signaling of PDAC in vivo. **a** Expressions of CDK1, p-STAT3 (Y705), CD44 and Sox2 in allograft tumors of KPC mice were examined by immunohistochemistry. Scale bars 200 μm. **b** Model indicating that regulation of epigenetic modification of CDK1–STAT3 signaling by fisetin leads to inhibition of cancer stemness properties in pancreatic cancer

To determine the in vivo efficacy of the combination regimen of fisetin and gemcitabine, we constructed a mouse allograft by transplanting primary pancreatic cancer tissue from KPC mice (Additional file 1: Figure S9b). Notably, tumor sizes were significantly reduced in fisetin- or gemcitabine-treated mice, with the combination regimen showing the best effect (Fig. 7g). The tumor weights were 4.145 ± 1.134 g, 1.181 ± 0.560 g, 0.834 ± 0.773 g, and 0.466 ± 0.384 g (mean ± standard deviation) in the control, fisetin, gemcitabine, and combination therapy groups, respectively, which suggested that combination therapy was significantly effective for pancreatic cancer in this mouse model (Fig. 7h). Moreover, immunohistochemical analysis of mice tumors demonstrated that CDK1, p-STAT3, CD44, and Sox2 were downregulated following fisetin alone or combination therapy treatment*,* which indicated that inhibition of the CDK1–STAT3 axis by fisetin contributed to enhance chemosensitivity in vivo (Fig. 8a). Together, these results confirmed that fisetin enhanced the effect of gemcitabine by reducing cancer stemness in vivo and in vitro (Fig. 8b).

## Discussion

PDAC is one of the most aggressive cancer types with poor survival outcomes, and the 5-year relative survival rate for patients with all clinical stages of pancreatic cancer combined is 9% [1–3]. This poor survival is attributable to low rates of early diagnosis and resistance to current chemotherapies, which might accumulate cancer stemness in various tumors [4–7]. Thus, novel therapeutic regimens are needed to restore drug sensitivity and improve patient survival by targeting PCSCs.

As a major member of the CDK family, CDK1 plays a dominant role in tumor initiation and stemness maintenance [10–12]. CDK1 orchestrates a dual mechanism by directly engaging with the pivotal cancer stemness-related protein Sox2 to drive tumor initiation in melanoma, while simultaneously preserving tumor stemness characteristics and pluripotency through phosphorylating TFCP2L1 in human bladder cancer [10, 11]. The present findings indicate that CDK1 might have a critical role in tumor stemness through its kinase function to catalyze the transfer of phosphate groups from ATP to target proteins. Here, we discovered that silencing *CDK1* significantly inhibited a subgroup of CD44+/CD24+ cells as well as the tumor stemness of pancreatic cancer.

Fisetin is a natural bioactive flavonoid that has anti-tumor properties in various tumor types [13]. Our previous studies found that fisetin shows anti-proliferation functions in human PDAC cells and improves the effect of the commonly used chemotherapy drug gemcitabine in vitro [14, 15]. Other studies have revealed that pancreatic cancer cells accumulate drug resistance against gemcitabine to augment cancer stem-like cells [16, 17]. Here, we further demonstrated that fisetin not only reduces the subgroups of CD44+/CD24+ cells that would be augmented by gemcitabine, but also has favorable synergistic effects in vitro and in vivo. Our studies revealed that these functions of fisetin are achieved mainly by regulating CDK1.

Our studies further demonstrated the effect of CDK1 in the maintenance of tumor stemness by mediating the phosphorylation of the important transcription factor STAT3. STAT3 is a critical transcription factor for maintaining tumorigenesis and tumor initiation via regulating the transcription of genes involved in the preservation of the stem cell phenotype in multiple cancer types such as glioma, prostate cancer, breast cancer, and liver cancer [25–28]. As for pancreatic cancer, STAT3 contributes to KRAS-induced PDAC initiation and progression, and inhibition of JAK–STAT3 signaling results in the impairment of a progenitor cell phenotype and abrogation of tumor sphere formation capacity [29–31]. STAT3 interacts with the promoter of pluripotent stem cell genes *SOX2*, *BIM1*, and *NANOG* to govern the transcription of these critical factors [17]. We identified that CDK1 activates STAT3 to regulate tumor sphere formation and the expression of stemness-related genes *CD44* and *SOX2*, as well as both CDK1 and STAT3, which are negatively correlated with the prognosis of PDAC patients.

PTMs including acetylation, ubiquitination, and phosphorylation have numerous roles in modulating multiple signaling pathways across diverse tumor types [32, 33]. There is increased evidence for the comprehensive crosstalk between PTMs; for example, acetylation of HEC1 at K53 and K59 sites weakens the phosphorylation of the N-terminal HEC1 region [32, 33]. Using acetyl-proteomics analysis and phospho-array assay, we confirmed that fisetin not only reduces CDK1 expression, but increases the acetylation of CDK1 at K33, consequently leading to the suppression of phosphorylation of CDK1 at Y15 in PDAC cells. Here, we determine that phosphorylation of CDK1 is critical for activating STAT3, which is achieved by phosphorylating STAT3 at Y705 site.

The evolutionarily conserved residue K33 is located in the catalytic pocket of CDK1, where it is essential for binding ATP; and lysines of similar active sites in other CDKs are involved in kinase activity [25, 34–36]. We found that mutation of K33 of CDK1 impaired its kinase capacity to activate STAT3; meanwhile, acetylation of K33 of CDK1 also weakened the phosphorylation of STAT3 at Y705. Moreover, we demonstrated that CDK1-wildtype interacted with STAT3, while mutations CDK1-K33R or CDK1-K33Q both impeded the interaction with STAT3, resulting in reduced phosphorylation of STAT3, which caused the diminishment of cancer stemness. In agreement with this, mutation or acetylation of other CDKs such as CDK2 at lysine 33 inhibit its kinase activity and affect cyclin A/CDK2 or cyclin E/CDK2 complexes [37].

We also found that HDAC3 as a deacetylase exhibits the ability to modulate the acetylation of CDK1 at K33, accompanying the changes in activity of CDK1 and STAT3. It has been discovered that HDAC3 is associated with self-renewal of liver CSCs and neural stem cells [22, 38]. HDAC3 is highly expressed in liver cancer, and silencing *HDAC3* inhibits both the proliferation and self-renewal of liver CSCs [38]. It was found that a lack of HDAC3 decreases the phosphorylation of STAT3 at Y705 in liver cancer and multiple myeloma [23, 24]. However, research into the relationship between HDAC3 and STAT3 is absent in human PDAC. Our data revealed that HDAC3 regulated the phosphorylation of STAT3 through the modulation of PTMs of CDK1 using its deacetylase function in PDAC. Interestingly, CDK1-K33Q showed a higher interaction capacity with HDAC3 than CDK1-WT, while CDK1-K33R impaired the interaction with HDAC3, suggesting that acetylated CDK1 is better able to bind HDAC3. HDAC3 expression also influenced cancer stemness in PDAC, supporting the opinion that HDAC3 contributes to drug resistance [39, 40]. Inhibition of HDAC3 is conducive to reversing the chemoresistance of leukemia cells and the MEKi resistance of PDAC cells [39, 40].

It is interesting that fisetin not only targets CDK1, but also reduces the expression of HDAC3 in PDAC cells, which indicates the potential of fisetin to sensitize PDAC cells to chemotherapy. To determine our hypothesis, we performed combination therapy in human and mouse PDAC cells and in an allograft model generated from KPC mice. Our data demonstrated that fisetin inhibited the cancer stemness induced by gemcitabine and enhanced the anti-tumor effect of gemcitabine for PDAC in vitro and in vivo*,* suggesting the potential of combination therapy of fisetin and gemcitabine for human PDAC.

In summary, CDK1 and STAT3, which are highly expressed in PDAC and PCSCs, are negatively correlated with the survival of PDAC patients and play an important role in maintaining pancreatic cancer stemness. Acetylation of CDK1 at K33 reduces pancreatic cancer stemness by inhibiting the phosphorylation of STAT3. Fisetin not only directly inhibits CDK1 but also decreases the expression of the deacetylase of CDK1, HDAC3, which results in acetylation of CDK1 at K33 and the impairment of phosphorylation of CDK1. This in turn causes downregulation of stemness-related genes and inhibition of PCSCs by inactivating STAT3. Our findings demonstrate that fisetin can sensitize PDAC cells to gemcitabine by suppressing PCSCs, which might provide an attractive therapeutic regimen for PDAC by targeting CSC-related genes.

## Methods

### Reagents and antibodies

Primary antibodies used in this study were as follows: anti-Actin (Abcam, ab179467), anti-CDK1 (Abcam, ab133327), anti-STAT3 (Abcam, ab109085; Abcam, ab32500), anti-CD44 (Abcam, ab50137), anti-CD24 (Abcam, ab179821), anti-Sox2 (Cell Signailing Technology, #23064; Abcam, ab92494), anti-Oct4 (Abcam, ab181557; Cell Signailing Technology, #2890), anti-Nanog (Cell Signailing Technology, #4903), anti-HDAC3 (Abcam, ab32369), anti-Myc-Tag (Cell Signailing Technology, #2276), anti-Phospho-CDK1 (Tyr15) (Abcam, ab47594; Cell Signailing Technology, #4539), anti-Phospho-CDK1 (Thr14) (Cell Signailing Technology, #2543); anti-Phospho-CDK1 (Thr161) (Cell Signailing Technology, #9114); anti-Phosoho-STAT3 (Tyr705) (Abcam, ab76315; Affinity, AF3293), anti-Phospho-STAT3 (Ser727) (Abcam, ab32143), anti-Acetyl-lysine (PTM Bio, PTM-105; PTM Bio, PTM-101; Cell Signaling Technology, #9441S), anti-Ubiquitin (Abcam, ab134953); FITC-CD44 (BD Biosciences, catlog.555478), PE-CD24 (BD Biosciences, catlog.555428), Fisetin was purchased from Selleck (catalog.S2298). Trichostatin A (TSA) and Nicotinamide (NAM) was purchased from MCE (catalog. HY-15144; catalog. HY-B0150).

### Cell culture

The human pancreatic cancer cell lines HPC-Y5, PANC-1 were obtained from the Stem Cell Bank, Chinese Academy of Sciences (Shanghai, China). PANC-1, HPC-Y5 cells were cultured in DMEM supplemented with 10% FBS. KPC203 cell line was generated from individual primary tumor derived from C57B1/6 KPC (Kras^LSL−G12D^ p53^LSL−R172H^ Pdx-1-Cre) mice at Zhejiang University. KPC203 cells were cultured in DMEM supplemented with 10% FBS, 2 mM l-glutamine, 1 mM non-essential amino acids, 1 mM sodium pyruvate, 20 mM HEPES, Gentamicin and Penicillin/Streptomycin. All cells were maintained in 37 °C and 5% CO_2_. As we previously reported [15], the concentration of fisetin was 100 µM for further investigation in pancreatic cancer cells.

### Proliferation assays

Cells were seeded in 96‐well plates with the treatment of fisetin (100 µM) or gemcitabine (10 µM), cells were scanned every one hours and cell confluence (%) was detected with an IncuCyte Live‐Cell imaging system (Essen BioScience, USA).

### Flow cytometry for CSC identification

To identify the ration of CSC, 1 × 10^5^ cells were stained for FITC-CD44, PE-CD24 (BD Biosciences). Gently vortex the cells and incubate for 30 min at room temperature in the dark. After staining with antibodies, cells were analyzed by flow cytometry.

### In situ proximity ligation assay

The in situ proximity ligation assay (PLA) was performed using Duolink kit (Sigma-Aldrich) with appropriate antibodies. Each red spot indicated a cluster of protein–protein interaction (the distance between two proteins is less than 40 nm), and distinct red spots were visualized with ZEIS LSM800 confocal fluorescence microscope. Co-localization quantification was calculated using Imaris software (https://imaris.oxinst.com/).

### Sphere formation assays

A total of 1 × 10^3^ cells were seeded in 24-well ultra-low cluster plates. Cell were cultured for 20 days in DMEM/F12 serum-free medium (Corning) supplemented with 2% B27 (Invitrogen), 20 ng/mL epidermal growth factor (EGF, PeproTech), 20 ng/mL basic fibroblast growth factor (bFGF, PeproTech), and 5 μg/mL insulin for sphere formation.

### Phospho-specific protein microarray analysis

Cell lysates were applied to the Phospho Explorer Antibody Array (Full Moon BioSystems, USA), and phospho-array detection and date analysis were performed by Wayen Biotechnology (Shanghai, China). A ratio computation was used to measure the extent of protein phosphorylation: phosphorylation ratio = phosphorylated value/unphosphorylated value.

### SILAC proteomics and acetyl-proteomics

#### SILAC (stable isotope labeling by amino acids in cell culture) Labeling

The PANC-1 cells were grown to 80% confluence in DMEM medium containing 10% fetal bovine serum and 1% penicillin–streptomycin at 37 °C with 95% air and 5% CO_2_. The control group was labeled with l-^13^C_6_-Lysine/l-^13^C_6_^15^N_4_-Arginine, and fisetin treated group was labeled with l-Lysine/l-Arginine using a SILAC Protein Quantitation Kit (Thermo, USA). The PANC-1 cells was cultured for more than six generations before being collected. Then cells were further grown in SILAC media to desired cell number.

#### Protein extraction and trypsin digestion

The labeled cells was collected and sonicated on ice using a high intensity ultrasonic processor in lysis buffer (8 M urea, 1% protease inhibitor cocktail). For acetylation experiments, 3 μM TSA and 50 mM NAM were also added to lysis buffer. The supernatant was collected by centrifugation at 12,000*g* at 4 °C for 15 min. For digestion, the protein solution was reduced with 5 mM dithiothreitol for 30 min at 56 °C and alkylated with 11 mM iodoacetamide for 15 min at room temperature in darkness. The protein sample was then diluted to urea concentration less than 2 M. Trypsin (trypsin: protein = 1:50) was added to protein sample for the first digestion overnight and 1:100 trypsin-to-protein mass ratio at 37 °C for a second 4 h-digestion.

#### HPLC fractionation and affinity enrichment

The peptides were separated by high pH reverse-phase HPLC on Thermo Betasil C18 column. The procedure was as follows: peptide was separated with gradient of 8%–32% acetonitrile (pH 9.0) over 60 min into 60 fractions. Then the peptides were combined into 4 components, and the combined components were freeze-dried by vacuum centrifuging. Tryptic peptides dissolved in NETN buffer (100 mM NaCl, 1 mM EDTA, 50 mM Tris–HCl, 0.5% NP-40, pH 8.0). To enrich acetylation modified peptides, samples were incubated with pre-washed acetyl-antibody beads (catlog: PTM-104, PTM Bio) at 4 °C overnight with gentle shaking. Then the acetyl-antibody beads were washed five times with NETN buffer and twice with deionized water. The bound peptides were eluted with 0.1% trifluoroacetic acid and freeze-dried by vacuum centrifuging. Finally, the peptides were desalted with C18 ZipTips (Millipore, USA) for LC–MS/MS analysis.

#### LC–MS/MS analysis

The peptides were dissolved in liquid chromatographic mobile phase A (0.1% (V/V) formic acid solution) and separated by ultra-high performance liquid chromatography system Easy-NLC 1000. The mobile phase B was an aqueous solution containing 0.1% formic acid and 90% acetonitrile. The gradient was comprised of an increase from 7 to 23% solvent B over 26 min, 23% to 35% for 8 min, and climbing to 80% in 3 min then holding at 80% for the last 3 min, all at a constant flow rate of 700 nL/min on an EASY-nLC 1000 UPLC system. The following analysis of peptides was carried out by Q Exactive™ Plus hybrid quadrupole-Orbitrap mass spectrometer (Thermo, USA).

### Molecular docking and MD simulation

Molecular docking between fisetin and CDK1 was performed using AutoDock Vina. The receptor site was prepared with PyMol using the CDK1 structure (PDB 4YC6) from the Protein Data Bank, as well as accessory protein CKS2 was removed. The grid box for docking was positioned at the catalytic pocket. A (250 ns) MD simulation was performed to investigate the functional mechanism of Fisetin on CDK1 by Desmond suit of Schrödinger LLC. The simulation systems were prepared by applying the OPLS4 force field to the complex and an dodecahedron box was generated by 10 Å border from the complexes. TIP3P water model was used to fill the box while sodium and chloride ions were added to a concentration of 0.15 M NaCl, and then additional ions were added to achieve charge neutrality(the other parameters were set up by default).

### GTEx and TCGA survival and tissue expression analyses

The expression of CDK1 and STAT3 in tumor-adjacent tissue and pancreatic cancer tissues, as well as survival analysis of CDK1 and STAT3, was determined based on RPKM values using GEPIA database [41].

### Immunohistochemistry

Immunohistochemical (IHC) staining was performed using the standard streptavidin–biotin–peroxidase complex method according to previously described protocols [14]. Mouse IHC analysis was performed to determine CDK1, p-STAT3-Y705, CD44 and Sox2 expression in pancreatic cancer tissues with treatment. The sections were observed under an Olympus CX31 microscope (Olympus, Japan). Cells with the cytoplasm stained yellow to brown were scored as immuno-positive.

### Transfection and siRNA

EP300-core, Flag-HDAC3, CDK1-WT-myc, CDK1-K33R-myc, CDK1-K33Q-myc, STAT3-WT-myc were constructed in pEGFP-N3-Vectors. The plasmids were transfected into cells using Lipofectamine 3000 (Invitrogen, USA). *CDK1, STAT3* and *HDAC3* siRNAs (Ribobio, China) were used to knock down *CDK1, STAT3* and *HDAC3* gene expression. The *CDK1, STAT3, HDAC3* and control siRNA (100 pM) were transfected into PANC-1, HPC-Y5 cells using siRNA transfection reagent (Ribobio, China) 24h before fisetin treatment. The cells were collected after fisetin treatment for 48h. The shRNA of *CDK1 *(*5ʹ-GTGGAATCTTTACAGGACTAT-3ʹ*) was constructed in pLKO.1 vector for stable down-regulating CDK1 in PANC-1 cells. Infected cell populations were selected using 2 mg/mL puromycin.

### Animals

Nude mice were obtained from SLAC Laboratory and bred in the animal center of Zhejiang University under specific pathogen-free conditions. Allograft PDAC model was established with tumor tissue derived from C57B1/6 KPC (KrasLSL-G12D p53LSL-R172H Pdx-1-Cre) mice. KPC mice was generated by Shanghai Model Organisms Centre (Shanghai, China). Tumor growth and the condition of mice were monitored every week. Control group animals were treated with DMSO via intraperitoneal injection. Treatment group mice were treated with fisetin (160 mg/kg body weight i.p.) alone, gemcitabine (50 mg/kg) alone or combination therapy every other day. Animal experiments were approved and performed in accordance with the institutional guidelines for animal care of the animal ethics committee of Zhejiang University.

### Immunofluorescence via confocal microscopy

Cells were fixed with 4% paraformaldehyde at room temperature for 15 min and then washed with PBS in 5 min for three times. Fixed cells were blocked in antibody dilution buffer (PBS containing 0.25% Triton X-100 and 1% bovine serum albumin). The cells were incubated with primary antibody at 4 °C overnight, and subsequently with Alexa Fluor 488-conjugated donkey anti-rabbit secondary antibody (Invitrogen, USA) for 1 h at room temperature. The nucleus was stained by incubation with DAPI (Abcam, USA) for 15 min at room temperature. Finally, Cells were scanned using a ZEISS LSM 880 confocal microscope (Zeiss, GER).

### Ethics statement

The research protocol was reviewed and approved by the Research Ethics Committee of Sir Run Run Shaw Hospital, School of Medicine, Zhejiang University. Animal experiments were approved and performed in accordance with the institutional guidelines for animal care of the animal ethics committee of Zhejiang University.

### Statistical analysis

Data were analyzed using SPSS version 22.0. Combinational index (CI) values were calculated using Compusyn software to confirm synergy [42]. CI < 1 indicates synergistic effects, CI = 1 indicates the mean additive effect of the drugs, and CI > 1 represents an antagonistic effect. Statistically significant differences within different experiments were determined using Student *t* test, and P < 0.05 was considered statistically significant.

### Supplementary Information

**Additional file 1: Figure S1. a** Representative flow cytometry plots for CD44 and CD24 expression in human pancreatic cancer HPC-Y5 cells with DMSO or fisetin treatment. Cells were treated with fisetin (100 µM) for 48 h. **b** Statistical plot of ratio of CD44 + /CD24 + positive and CD44-/CD24- negative cells in control or fisetin treatment HPC-Y5 cells. Data are presented as mean ± SD (n = 3); *P < 0.05. **c** Number of differentially expressed proteins quantified by SILAC proteomics analysis after fisetin treatment in PANC-1 cells, which were divided into four quantiles (Q1-Q4) according to their ratios of fold change: Q1 (0 < Ratio ≤ 1/1.5), Q2 (1/1.5 < Ratio ≤ 1/1.2), Q3 (1.2 < Ratio ≤ 1.5) and Q4 (Ratio > 1.5). **d** Heat map of Biological Process in GO enrichment analysis of differentially expressed proteins in each Q subset according to P value of Fisher's exact test. **Figure S2.** Heat map of Cellular Component in GO enrichment analysis of differentially expressed proteins in each Q subset according to P value of Fisher's exact test. **Figure S3.** Heat map of Molecular Function in GO enrichment analysis of differentially expressed proteins in each Q subset according to P value of Fisher's exact test. **Figure S4. a** Heat map of KEGG pathway enrichment analysis of differentially expressed proteins in each Q subset according to P value of Fisher's exact test. Enrichment pathways of Q1 and Q2 indicated proteins in important pathways including PI3K–Akt signaling, pathways in cancer, metabolism pathways and ECM-receptor interaction were declined in PANC-1 cells with fisetin treatment. **b** Heat map of protein domain enrichment analysis of differentially expressed proteins in each Q subset. Enrichment protein domain of Q1 and Q2 indicated proteins with EGF-like domain and Laminin EGF domain were reduced by fisetin treatment. **c** Protein domain enrichment analysis of whole differentially expressed proteins quantified by proteomics analysis. **Figure S5. a** Summary of acetylated sites and proteins quantified by acetyl-proteomics analysis. **b** Summary of differentially expressed acetylated sites and proteins quantified by acetyl-proteomics analysis. 368 sites were changed over 1.2-folds (P < 0.05) including 307 up-regulated and 61 down-regulated in 264 proteins. **c** Go enrichment analysis of differentially expressed acetylated proteins. **d** Immunoprecipitation and western blot determined that EP300 was acetyl-transferase of CDK1. **Figure S6.** Protein motif analysis was performed by statistical analysis of the patterns of amino acid sequences before and after all acetylated sites in samples. 19 types of conserved motifs were identified by motif analysis. **Figure S7. a** Western blot analysis was used to determine expression of CDK1, STAT3, CD44 and Sox2 in adherent PANC-1 cells or spheres generated from PANC-1 cells. Adhe, adherent cells. **b** Inhibition of CD44 and Sox2 by CDK1 silencing can be rescued by over-expressing STAT3. Expression of CD44, Sox2, CDK1, STAT3, p-CDK1 and p-STAT3 were examined by western blot analysis. **c** Inhibition of CDK1-STAT3 signaling by fisetin in purified pancreatic cancer stem cell spheres. Expression of CD44, Sox2, CDK1, STAT3, p-CDK1 and p-STAT3 were performed by western blot analysis. **d-e** Direct suppression of purified pancreatic oncospheres by fisetin. Second generation of PANC-1 and HPC-Y5 cells from oncospheres were subjected to the tumor sphere-formation assay in ultra-low cluster plates. After the cultivation process to initial point, these tumor spheres were treated with or without fisetin (100 μM) for 48 h. Scale bars, 100 μm. Data are presented as mean ± SD (n = 3),*P < 0.05; ns, no significance. **Figure S8. a** HDAC3 silencing reduced levels of p-CDK1, p-STAT3, CD44 and Sox2. Western blot analysis was used to determine expression of CDK1, STAT3, CD44 and Sox2 in HDAC3 silencing PANC-1 cells. **b-c** Lack of HDAC3 weakened tumor sphere formatting capacity in PDAC cells. Scale bars, 100 μm. Data are presented as mean ± SD (n = 4); *P < 0.05. **d** HDAC3 over-expression increased levels of p-CDK1, p-STAT3, CD44 and Sox2. Western blot analysis was used to determine expression of CDK1, STAT3, CD44 and Sox2 in HDAC3 over-expression PANC-1 cells. **e–f** Over-expressing HDAC3 enhanced the sphere formatting capacity both in PANC-1 and HPC-Y5 cells. Scale bars, 100 μm. Data are presented as mean ± SD (n = 4); *P < 0.05, **P < 0.01. **g** Fisetin reduced expression of HDAC3 both in PANC-1 and HPC-Y5 cells. **Figure S9. a** Fraction-affected (Fa) and CI are explored after 48-h incubation with fisetin and gemcitabine combination, CI < 1 represents synergy. **b** Representative section of magnetic resonance imaging (MRI) for spontaneous pancreatic ductal adenocarcinoma in KPC mice. **Figure S10. a** Western blot analysis was used to determine expression of CDK1 in stable CDK1 knockout PANC-1 cells. CDK1-KO: CDK1-knockout. **b** Quantification of relative phosphorylation of CDK1 was performed by analyzing western blot results with image J software. The relative phosphorylation of CDK1 was significant reduced in PANC-1 and HPC-Y5 cells with fisetin (100 μM) treatment. Fis, fisetin; *P < 0.05; ns, no significance. **c** qRT-PCR showed that mRNA expression of CDK1 was not influenced by fisetin treatment in pancreatic cancer cells. Data are presented as mean ± SD (n = 3). ns, no significance. **d** Proteasome inhibitor MG132 restore the expression of CDK1 in PDAC cells with fisetin treatment. PANC-1 and HPC-Y5 ells were cultured with or without fisetin (100 μM) treatment for 48 h. Before the collection of cells, MG132 (10 μM) were added to culture medium for 0, 6 and 12 h. h, hours. **e** Immunoprecipitation and western blot determined that fisetin induced prominent ubiquitination of CDK1 in pancreatic cancer cells. IP, immunoprecipitation. Ub, ubiquitin. **Figure S11. a** Western blot analysis was used to determine phosphorylation of CDK1 at Thr14 and Thr161 residues in pancreatic cancer PANC-1 and HPC-Y5 cells. p-CDK1(T161): phosphorylation of CDK1 at Thr161; p-CDK1(T14): phosphorylation of CDK1 at Thr14.

**Additional file 2: Table S1.** Proteins identified by proteomic.

**Additional file 3: Table S2.** Protein kinase like domain associated genes.

**Additional file 4: Table S3.** Levels of transcriptome and protein phosphorylation of genes in HIF-1 signaling.

**Additional file 5: Table S4.** Levels of transcriptome and protein phosphorylation of genes in JAK–STAT signaling.

## Data Availability

Original data from RNA-Seq are available in the Gene Expression Omnibus (GEO; https://www.ncbi.nlm.nih.gov/geo/; Accession Numbers: GSE117189). The authors declare that all data supporting the findings of this study are available within the paper in the main text or Additional files.
