# Supplementary material for: Post-translational modification of CDK1–STAT3 signaling by fisetin suppresses pancreatic cancer stem cell properties
Source: Cell Biosci. 2023 Sep 24;13:176. doi: 10.1186/s13578-023-01118-z (PMC10518106; doi:10.1186/s13578-023-01118-z)
Supplement: Supplementary file 1 — Additional file 1: Figure S1. a Representative flow cytometry plots for CD44 and CD24 expression in human pancreatic cancer HPC-Y5 cells with DMSO or fisetin treatment. Cells were treated with fisetin (100 µM) for 48 h. b Statistical plot of ratio of CD44 + /CD24 + positive and CD44-/CD24- negative cells in control or fisetin treatment HPC-Y5 cells. Data are presented as mean ± SD (n = 3); *P < 0.05. c Number of differentially expressed proteins quantified by SILAC proteomics analysis after fisetin treatment in PANC-1 cells, which were divided into four quantiles (Q1-Q4) according to their ratios of fold change: Q1 (0 < Ratio ≤ 1/1.5), Q2 (1/1.5 < Ratio ≤ 1/1.2), Q3 (1.2 < Ratio ≤ 1.5) and Q4 (Ratio > 1.5). d Heat map of Biological Process in GO enrichment analysis of differentially expressed proteins in each Q subset according to P value of Fisher's exact test. Figure S2. Heat map of Cellular Component in GO enrichment analysis of differentially expressed proteins in each Q subset according to P value of Fisher's exact test. Figure S3. Heat map of Molecular Function in GO enrichment analysis of differentially expressed proteins in each Q subset according to P value of Fisher's exact test. Figure S4. a Heat map of KEGG pathway enrichment analysis of differentially expressed proteins in each Q subset according to P value of Fisher's exact test. Enrichment pathways of Q1 and Q2 indicated proteins in important pathways including PI3K–Akt signaling, pathways in cancer, metabolism pathways and ECM-receptor interaction were declined in PANC-1 cells with fisetin treatment. b Heat map of protein domain enrichment analysis of differentially expressed proteins in each Q subset. Enrichment protein domain of Q1 and Q2 indicated proteins with EGF-like domain and Laminin EGF domain were reduced by fisetin treatment. c Protein domain enrichment analysis of whole differentially expressed proteins quantified by proteomics analysis. Figure S5. a Summary of acetylated sites and protei [file 13578_2023_1118_MOESM1_ESM.docx]

**Additional Information**

**Post-****translational modification of CDK1–STAT3 signaling by fisetin suppresses pancreatic cancer stem cell properties**

Xiaodong Xu^1,4#^, Yimin Ding^1#^, Junbin Jin^1^, Chengjie Xu^1^, Wenyi Hu^1^, Songtao Wu^1^, Guoping Ding^1^, Rui Cheng^3^, Liping Cao^1,2*^, Shengnan Jia^1,2*^

1.Department of General Surgery, Sir Run Run Shaw Hospital, School of Medicine, Zhejiang University, Hangzhou 310000, China.

2.Innovation Center for Minimally Invasive Technique and Device, Zhejiang University, Hangzhou, Zhejiang, 310000, China.

3.State Key Laboratory of Food Science and Technology, Jiangnan University, 1800 Lihu Avenue, Wuxi 214122, China

4.General Surgery, Cancer Center, Department of Colorectal Surgery, Zhejiang Provincial People's Hospital (Affiliated People's Hospital, Hangzhou Medical College), Hangzhou, Zhejiang, 310014, China.

*Corresponding author: Prof. Liping Cao.Tel: +86 0571 86090073; Fax: +86 0571 86090073; Email: [caolipingzju@zju.edu.cn](mailto:caolipingzju@zju.edu.cn) PhD. Shengnan Jia. Email: [jiashengnan8180@zju.edu.cn](mailto:dinguop@zju.edu.cn)

**#These authors contributed equally to this work.**

**
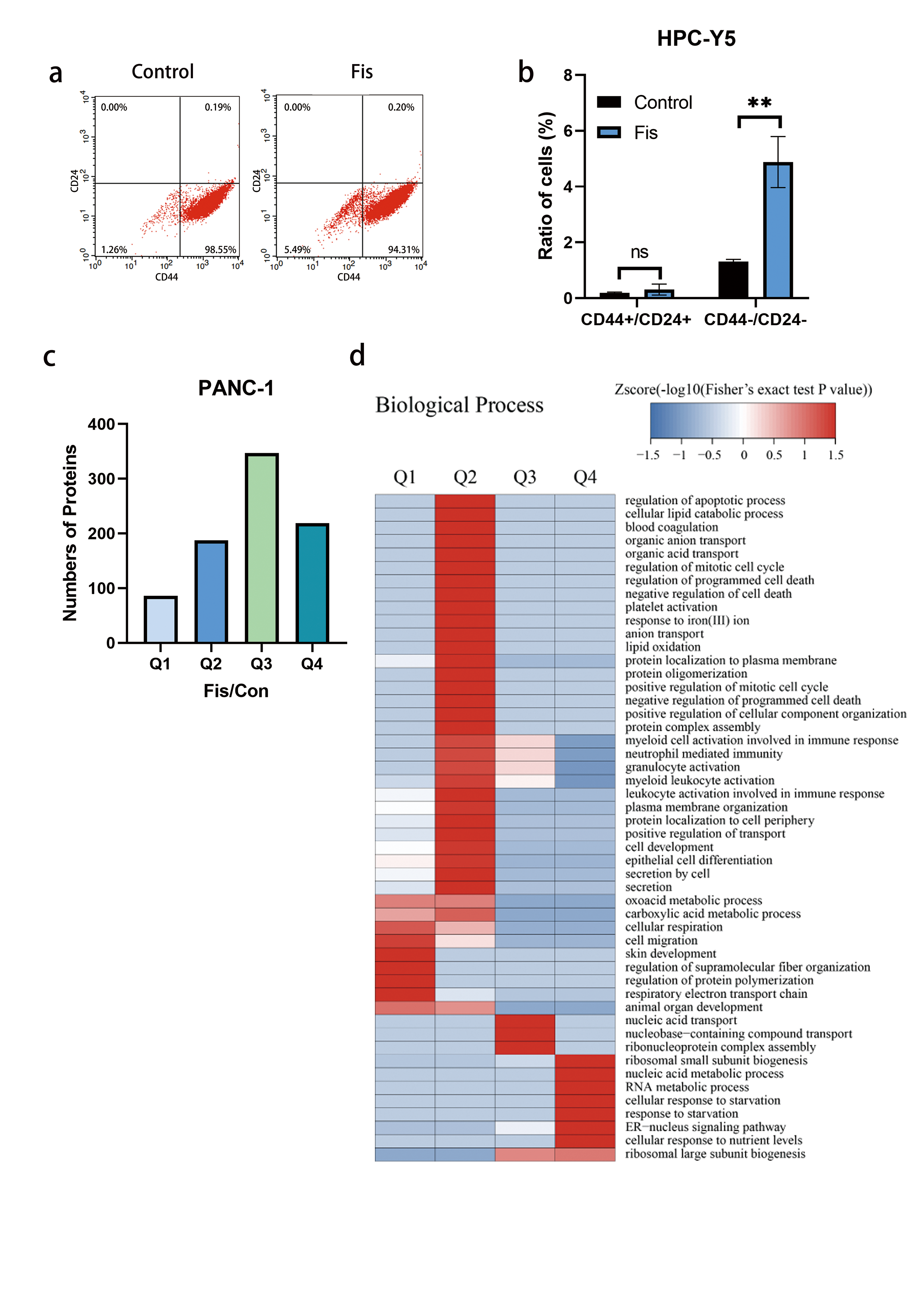
**

**Figure S1**

**a** Representative flow cytometry plots for CD44 and CD24 expression in human pancreatic cancer HPC-Y5 cells with DMSO or fisetin treatment. Cells were treated with fisetin (100µM) for 48h. **b** Statistical plot of ratio of CD44+/CD24+ positive and CD44-/CD24- negative cells in control or fisetin treatment HPC-Y5 cells. Data are presented as mean ± SD (n=3); *P<0.05. **c** Number of differentially expressed proteins quantified by SILAC proteomics analysis after fisetin treatment in PANC-1 cells, which were divided into four quantiles (Q1-Q4) according to their ratios of fold change: Q1 (0< Ratio ≤ 1/1.5), Q2 (1/1.5 < Ratio ≤ 1/1.2), Q3 (1.2 < Ratio ≤ 1.5) and Q4 (Ratio >1.5). **d** Heat map of Biological Process in GO enrichment analysis of differentially expressed proteins in each Q subset according to P value of Fisher's exact test.


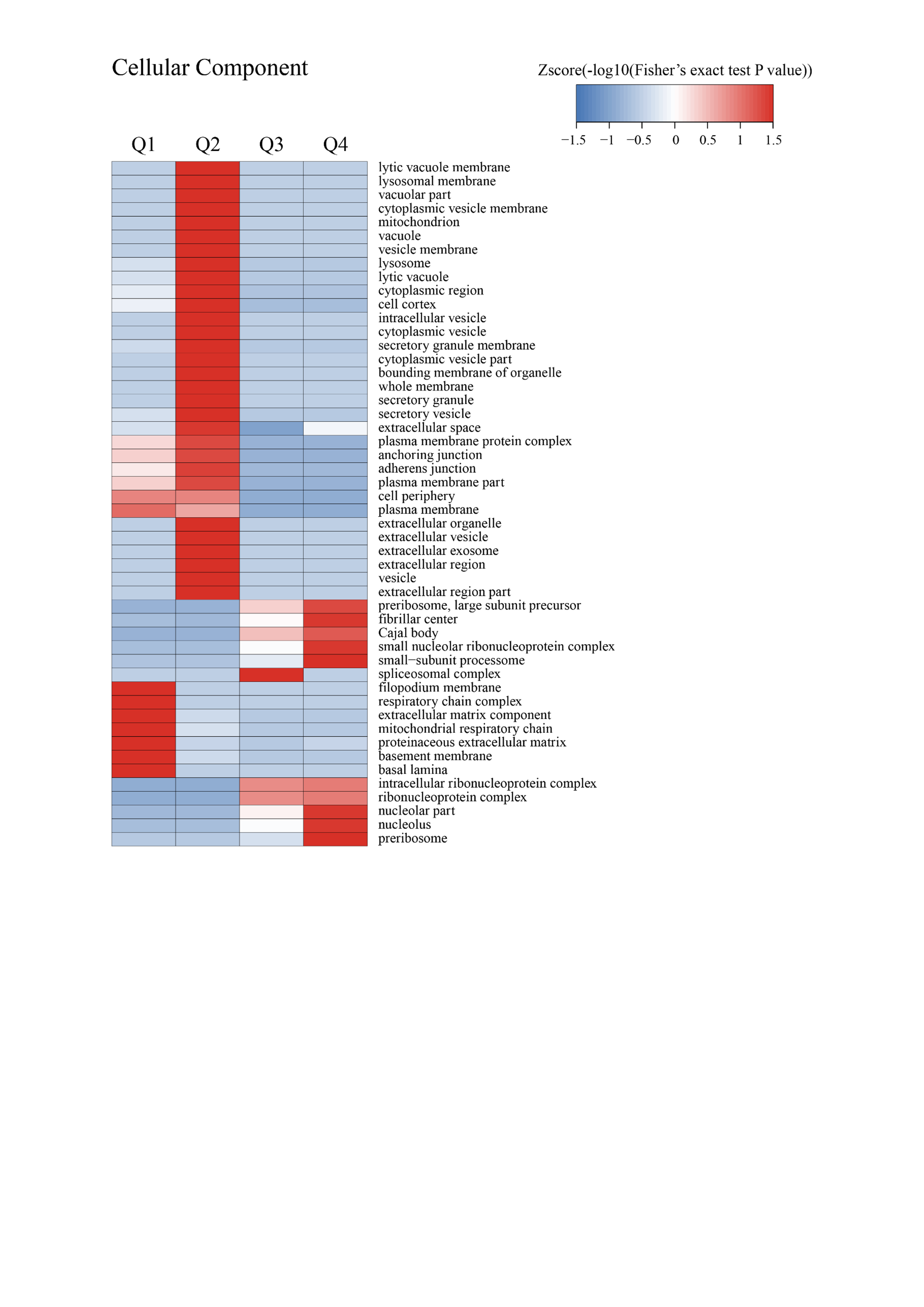


**Figure S2**

Heat map of Cellular Component in GO enrichment analysis of differentially expressed proteins in each Q subset according to P value of Fisher's exact test.


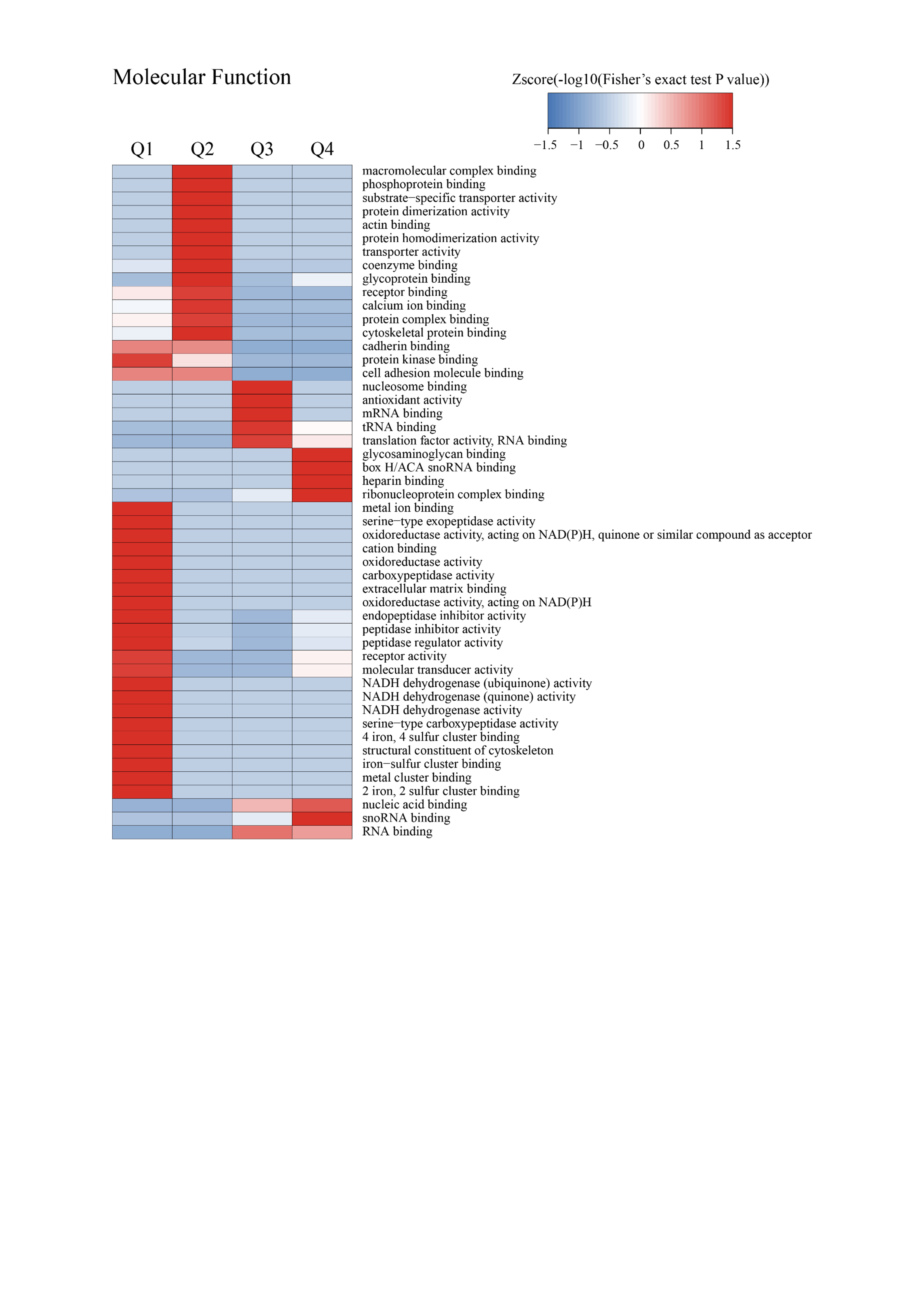


**Figure S3**

Heat map of Molecular Function in GO enrichment analysis of differentially expressed proteins in each Q subset according to P value of Fisher's exact test.


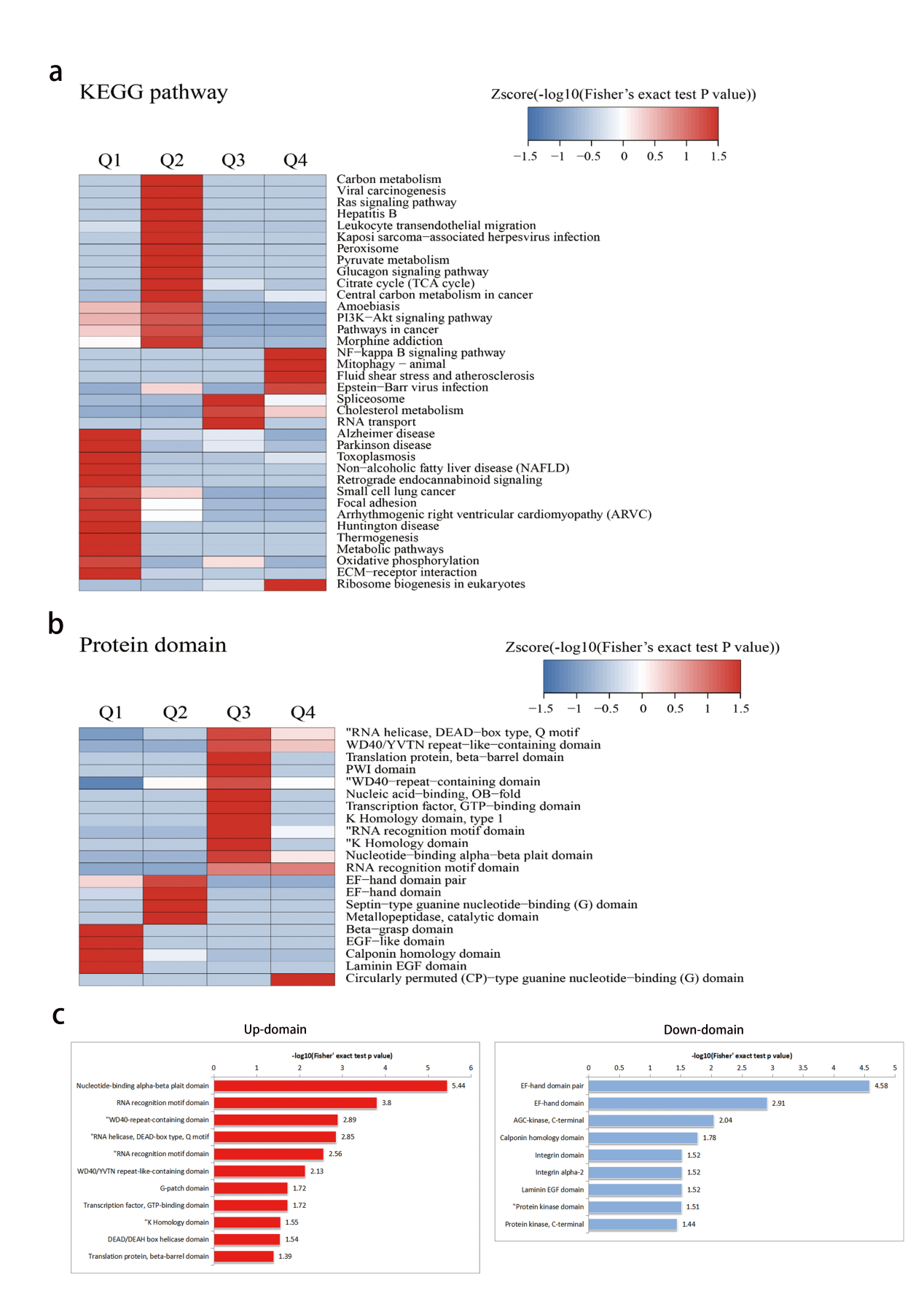


**Figure S4**

**a** Heat map of KEGG pathway enrichment analysis of differentially expressed proteins in each Q subset according to P value of Fisher's exact test. Enrichment pathways of Q1 and Q2 indicated proteins in important pathways including PI3K-Akt signaling, pathways in cancer, metabolism pathways and ECM-receptor interaction were declined in PANC-1 cells with fisetin treatment. **b** Heat map of protein domain enrichment analysis of differentially expressed proteins in each Q subset. Enrichment protein domain of Q1 and Q2 indicated proteins with EGF-like domain and Laminin EGF domain were reduced by fisetin treatment. **c** Protein domain enrichment analysis of whole differentially expressed proteins quantified by proteomics analysis.

**
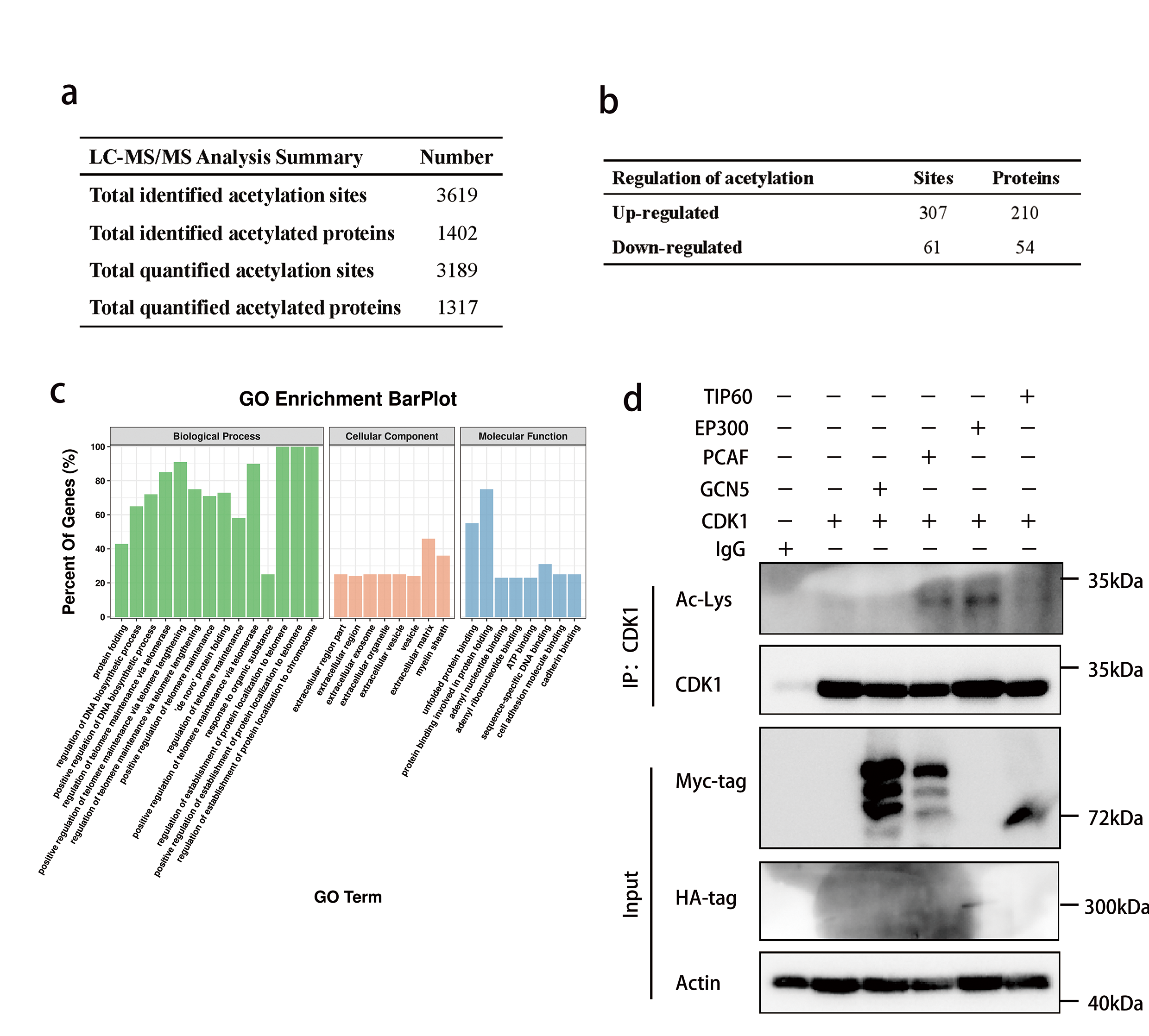
**

**Figure S5**

**a** Summary of acetylated sites and proteins quantified by acetyl-proteomics analysis. **b** Summary of differentially expressed acetylated sites and proteins quantified by acetyl-proteomics analysis. 368 sites were changed over 1.2-folds (P<0.05) including 307 up-regulated and 61 down-regulated in 264 proteins. **c** Go enrichment analysis of differentially expressed acetylated proteins. **d** Immunoprecipitation and western blot determined that EP300 was acetyl-transferase of CDK1.

**
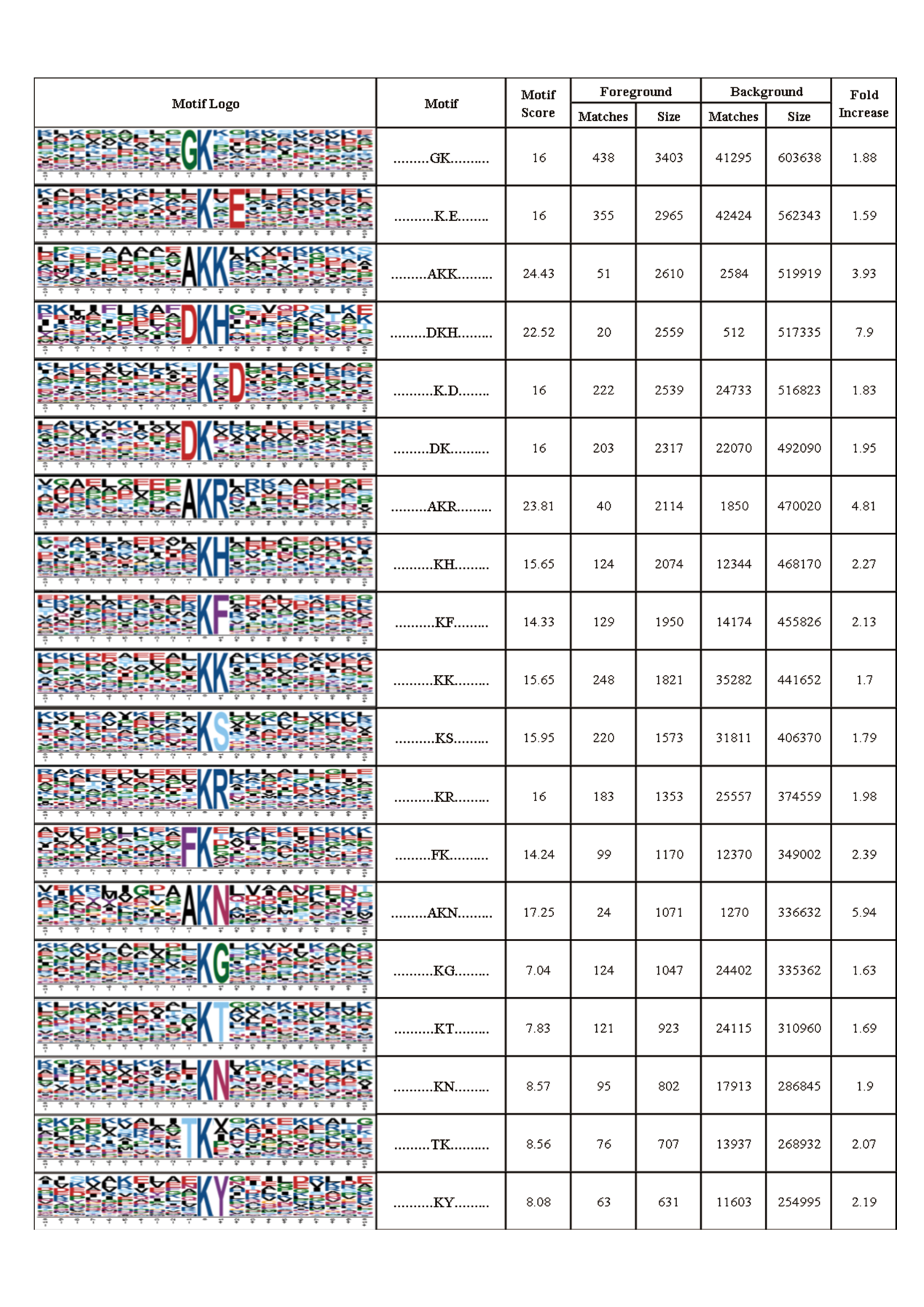
**

**Figure S6**

Protein motif analysis was performed by statistical analysis of the patterns of amino acid sequences before and after all acetylated sites in samples. 19 types of conserved motifs were identified by motif analysis.

**
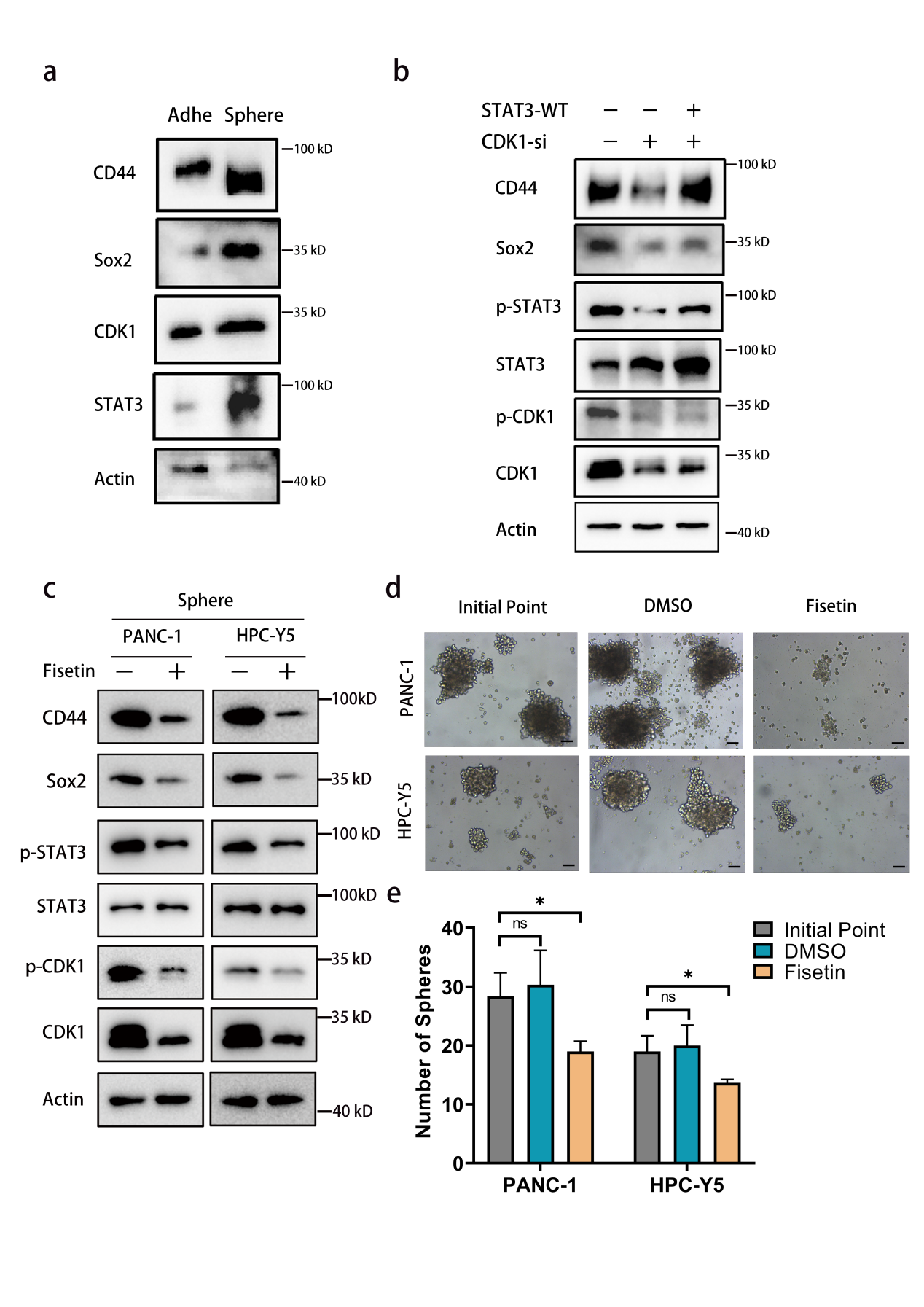
**

**Figure S7**

**a** Western blot analysis was used to determine expression of CDK1, STAT3, CD44 and Sox2 in adherent PANC-1 cells or spheres generated from PANC-1 cells. Adhe, adherent cells. **b** Inhibition of CD44 and Sox2 by CDK1 silencing can be rescued by over-expressing STAT3. Expression of CD44, Sox2, CDK1, STAT3, p-CDK1 and p-STAT3 were examined by western blot analysis. **c** Inhibition of CDK1-STAT3 signaling by fisetin in purified pancreatic cancer stem cell spheres. Expression of CD44, Sox2, CDK1, STAT3, p-CDK1 and p-STAT3 were performed by western blot analysis. **d-e** Direct suppression of purified pancreatic oncospheres by fisetin. Second generation of PANC-1 and HPC-Y5 cells from oncospheres were subjected to the tumor sphere-formation assay in ultra-low cluster plates. After the cultivation process to initial point, these tumor spheres were treated with or without fisetin (100μM) for 48h. Scale bars, 100 μm. Data are presented as mean ± SD (n=3),*P<0.05; ns, no significance.


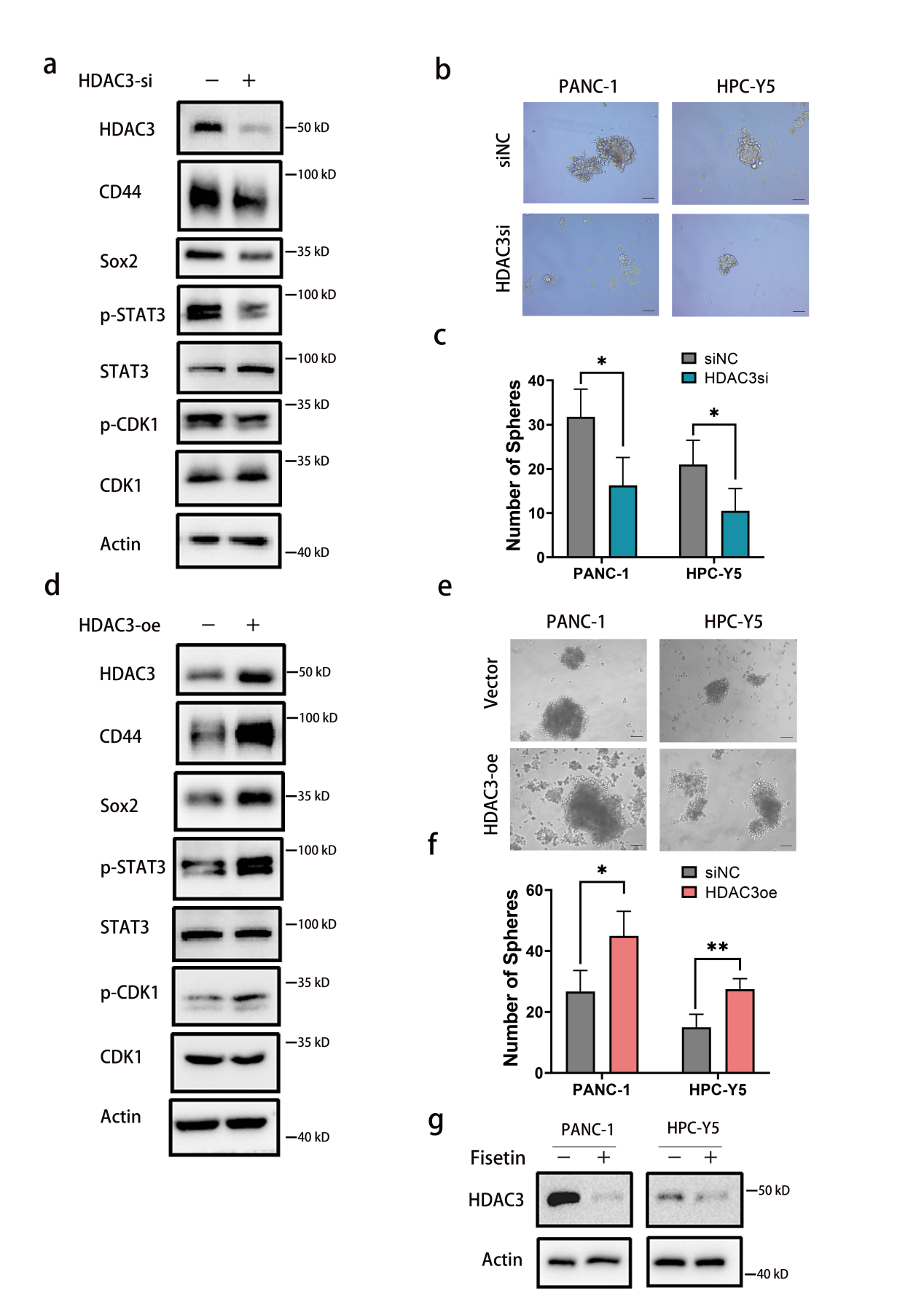


**Figure S8**

**a** HDAC3 silencing reduced levels of p-CDK1, p-STAT3, CD44 and Sox2. Western blot analysis was used to determine expression of CDK1, STAT3, CD44 and Sox2 in HDAC3 silencing PANC-1 cells. **b-c** Lack of HDAC3 weakened tumor sphere formatting capacity in PDAC cells. Scale bars, 100 μm. Data are presented as mean ± SD (n=4); *P<0.05. **d** HDAC3 over-expression increased levels of p-CDK1, p-STAT3, CD44 and Sox2. Western blot analysis was used to determine expression of CDK1, STAT3, CD44 and Sox2 in HDAC3 over-expression PANC-1 cells. **e-f** Over-expressing HDAC3 enhanced the sphere formatting capacity both in PANC-1 and HPC-Y5 cells. Scale bars, 100 μm. Data are presented as mean ± SD (n=4); *P<0.05, **P<0.01. **g** Fisetin reduced expression of HDAC3 both in PANC-1 and HPC-Y5 cells.


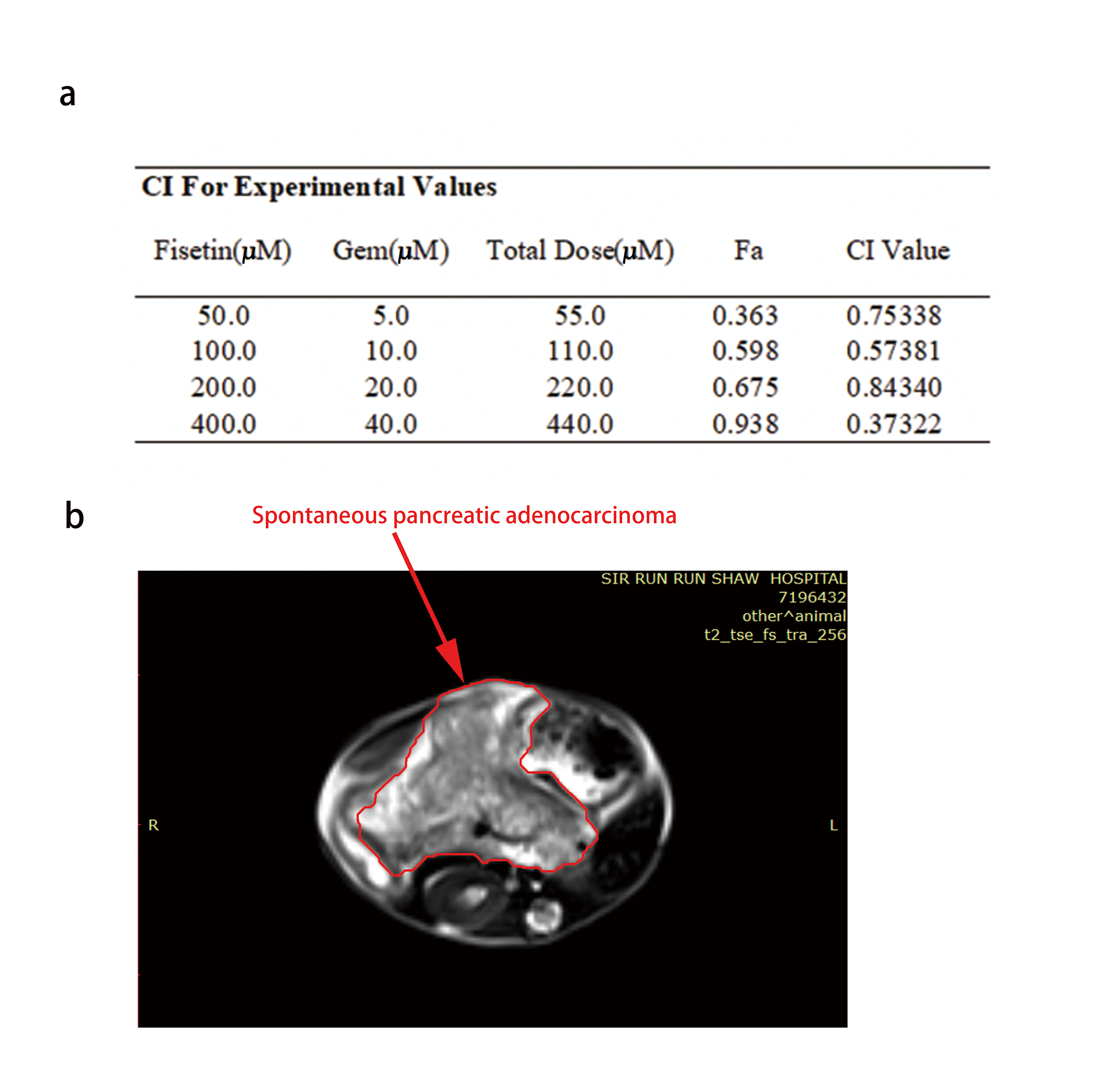


**Figure S9**

**a** Fraction-affected (Fa) and CI are explored after 48-hour incubation with fisetin and gemcitabine combination, CI<1 represents synergy. **b** Representative section of magnetic resonance imaging (MRI) for spontaneous pancreatic ductal adenocarcinoma in KPC mice.


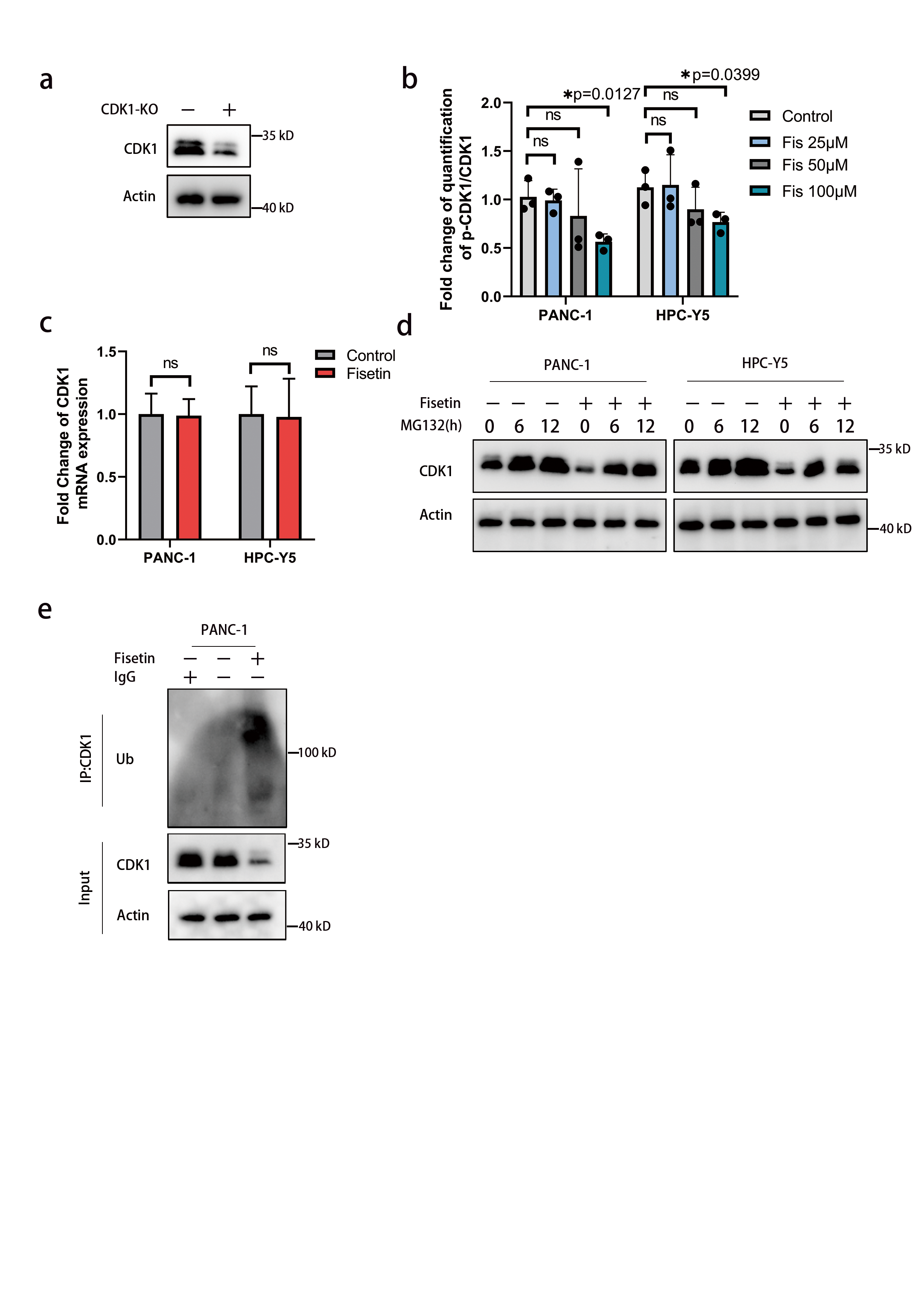


**Figure S10**

**a** Western blot analysis was used to determine expression of CDK1 in stable CDK1 knockout PANC-1 cells. CDK1-KO: CDK1-knockout. **b** Quantification of relative phosphorylation of CDK1 was performed by analyzing western blot results with image J software. The relative phosphorylation of CDK1 was significant reduced in PANC-1 and HPC-Y5 cells with fisetin (100 μM) treatment. Fis, fisetin; *P<0.05; ns, no significance. **c** qRT-PCR showed that mRNA expression of CDK1 was not influenced by fisetin treatment in pancreatic cancer cells. Data are presented as mean ± SD (n = 3). ns, no significance. **d** Proteasome inhibitor MG132 restore the expression of CDK1 in PDAC cells with fisetin treatment. PANC-1 and HPC-Y5 ells were cultured with or without fisetin (100 μM) treatment for 48h. Before the collection of cells, MG132 (10 μM) were added to culture medium for 0, 6 and 12 hours. h, hours. **e** Immunoprecipitation and western blot determined that fisetin induced prominent ubiquitination of CDK1 in pancreatic cancer cells. IP, immunoprecipitation. Ub, ubiquitin.


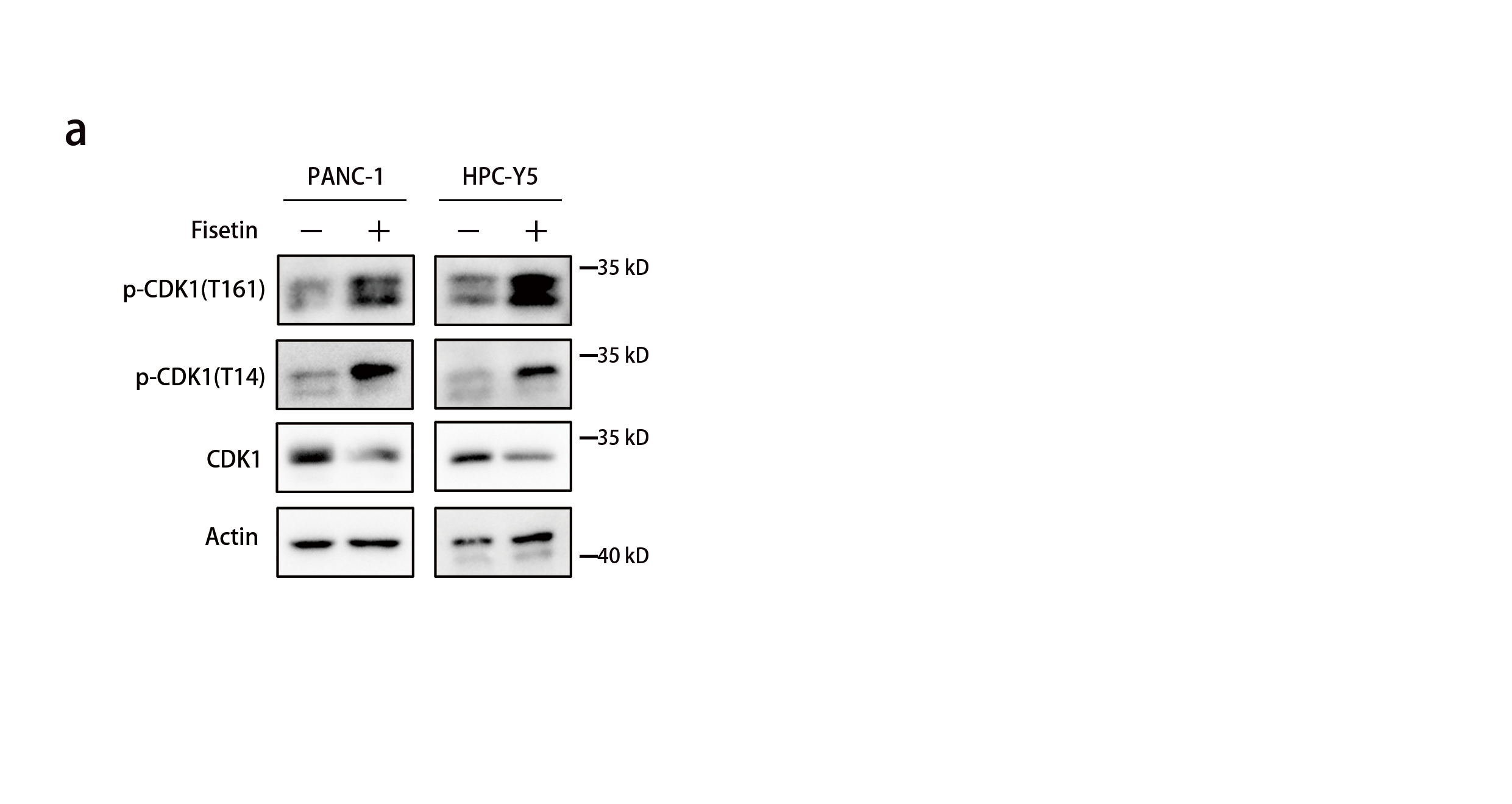


**Figure S11**

**a** Western blot analysis was used to determine phosphorylation of CDK1 at Thr14 and Thr161 residues in pancreatic cancer PANC-1 and HPC-Y5 cells. p-CDK1(T161): phosphorylation of CDK1 at Thr161; p-CDK1(T14): phosphorylation of CDK1 at Thr14.
